# Investigating the Potential of L(+)-Lactic Acid as a Green Inhibitor and Eradicator of a Dual-Species *Campylobacter* spp. Biofilm Formed on Food Processing Model Surfaces

**DOI:** 10.3390/microorganisms12112124

**Published:** 2024-10-23

**Authors:** Dimitra Kostoglou, Martha Apostolopoulou, Athina Lagou, Spyros Didos, Anagnostis Argiriou, Efstathios Giaouris

**Affiliations:** Department of Food Science and Nutrition, School of the Environment, University of the Aegean, 81400 Myrina, Lemnos, Greece

**Keywords:** *Campylobacter*, mixed-culture biofilms, polystyrene, stainless steel, aerobiosis conditions, disinfection, food safety, natural antimicrobials, untargeted metabolomics, public health

## Abstract

*Campylobacter* spp. are prevalent foodborne bacterial enteric pathogens. Their inclusion in biofilms on abiotic surfaces is considered a strategy that facilitates their extraintestinal survival. Organic acid (OA) treatments could be used in a green approach to decontaminate various surfaces. This work aimed to evaluate the inhibitory and eradicative effects of L(+)-lactic acid (LA), a naturally occurring OA, on a dual-species biofilm formed on two food processing model surfaces (polystyrene and stainless steel) by three selected foodborne *Campylobacter* spp. isolates (two *C. jejuni* and one *C. coli*). The influence of aerobiosis conditions (microaerophilic, aerobic and CO_2_ enriched) on the resistance of the established biofilms to the acid was also tested. In parallel, the predominant metabolites contained in the planktonic media of biofilm monocultures and mixed-culture biofilm were comparatively analyzed by an untargeted metabolomics approach. Results revealed that LA inhibited mixed-culture biofilm formation by more than 2 logs (>99%) on both surfaces when this was applied at its highest tested concentration (4096 μg/mL; 0.34% *v*/*v*). However, all the preformed mixed-culture biofilms (ca. 10^6−7^ CFU/cm^2^) could not be eradicated even when the acid was used at concentrations exceeding 5% *v*/*v*, denoting their extremely high recalcitrance which was still influenced by the abiotic substratum, and the biofilm-forming aerobiosis conditions. The metabolic analysis revealed a strain-specific metabolite production which might also be related to the strain-specific biofilm-forming and resistance behaviors and resulted in the distinct clustering of the different samples. Overall, the current findings provide important information on the effectiveness of LA against biofilm campylobacteria and may assist in mitigating their risk in the food chain.

## 1. Introduction

*Campylobacter* spp. are leading causes of bacterial gastroenteritis globally, with *C. jejuni* and *C. coli* being the two species most responsible for human infection [1]. In Europe, North America, and Australia, campylobacteriosis is consecutively, in recent years, the most notified foodborne bacterial infection [2]. In 2022, 137,107 confirmed cases of that disease were reported to the European Food Safety Authority (EFSA), with a case fatality ratio of 0.04% [3], whereas the World Health Organization (WHO) estimates that nearly 5 million cases occur every year in the European region [4]. Similarly, *Campylobacter* spp. are estimated to cause 1.5 million illnesses each year in the United States [5]. These microaerophilic and Gram-negative pathogens are primarily transmitted through the consumption of contaminated food, particularly undercooked poultry products and unpasteurized milk [1]. The effects of campylobacteriosis can greatly vary, from mild to severe cases of diarrheal disease [6]. Alarmingly, some severe post-infection complications like Guillain–Barré syndrome, reactive arthritis, and additional sequelae are occasionally observed in individuals who have recovered from campylobacteriosis [7]. 

Biofilms are microbial communities that adhere to surfaces or are even accumulated at interfaces (e.g., air-liquid) and are embedded in a hydrated extracellular polymeric material mainly produced by cells [8]. These represent the default microbial growth mode in most environments and are well-known to protect microorganisms from various stresses (e.g., extreme temperature and/or pH, harmful oxygen levels, high salinity, nutrient starvation, antimicrobials, immune system attack, etc.) through several mechanisms that may also sometimes act in parallel [9,10,11]. Interestingly, biofilm entrapment is considered a critical aspect of the survival and persistence of microaerophilic *Campylobacter* spp. in extraintestinal environments. This facilitates their transmission to new hosts and, in parallel, contributes significantly to the acquisition and spread of antimicrobial resistance (AMR) [12,13,14]. Thus, like several other bacterial species, *Campylobacter* spp. can form de novo biofilms on their own or be integrated into other biofilms formed by different species where they happen to be found in the same ecological niche on a variety of either abiotic or biotic surfaces encountered within the food production chain (from the farm to the fork) [15,16]. Within biofilms, *Campylobacter* cells may survive in otherwise lethal oxygen-enriched environments and exhibit increased tolerance to antibiotics and disinfectants compared to their planktonic counterparts, posing significant challenges to their effective control and eradication [17]. Alarmingly, intra- and inter-species interactions encountered within mixed-culture biofilms, also those including *Campylobacter* spp., can affect their physiology and may further increase their resilience and perseverance [18]. 

To fight *Campylobacter* spp., adopting cost-efficient and eco-friendly control and eradication methodologies is paramount for ensuring environmental sustainability, minimizing reliance on traditional chemical disinfectants, and fostering improved public health outcomes. In this framework, organic acid (OA) treatments may offer a promising option for decontaminating various surfaces, potentially targeting and eliminating unwanted campylobacteria [19,20,21]. The most common OAs are the carboxylic acids, whose acidity is associated with their carboxyl group (–COOH), such as acetic, lactic, formic, propionic, malic, and citric acids, and their primary modes of antimicrobial action involve cytoplasmic acidification, the disruption of energy metabolism, nucleic acid and protein damages, and interference with overall metabolic processes [22,23,24]. These are found in natural sources (e.g., fruits) and/or may be produced through fermentation from renewable materials and have been extensively studied, either alone or synergistically with other treatments, for their efficacy against a broad spectrum of foodborne pathogens [25,26,27,28], also including *Campylobacter* spp. [19,20,29,30,31,32]. 

Indeed, several OAs are already used as food preservatives [33], while lactic acid (LA; 2-hydroxypropionic acid), as well as acetic acid, are also primarily applied for the acid washing of carcasses in Europe and the USA to decrease their microbial load [34,35]. Moreover, OAs in general are assumed to protect poultry from pathogenic diseases by altering the pH of the gastrointestinal tract (GIT) and therefore changing the composition of the microbiome [36]. However, their effect on live birds’ more general performance remains controversial [31]. Lactic acid is a naturally occurring OA that is found in fermented foods (such as yogurt, sauerkraut, and pickles) and can be sufficiently recovered through the fermentation of several inexpensive substrates [37]. It is the simplest hydroxycarboxylic acid, is soluble in water and exists in two enantiomeric forms: L-(+)-LA and D-(−)-LA [38]. It is produced industrially by the fermentation of carbohydrates by lactic acid bacteria (LAB) strains and is widely used in the food, pharmaceutical, chemical, cosmetic, and healthcare industries, as well as to produce the biodegradable biocompatible polymer polylactic acid [39,40]. Even though both of its isomeric forms are used in industry, L-(+)-LA is preferred for medical applications because it can be assimilated by the human body [41]. As a food additive, it is approved in many countries and used as a food preservative, curing agent, and flavoring agent (ingredient E270) [42]. However, to the best of our knowledge, the studies on the application of it, as well as other OAs, against *Campylobacter* biofilm cells are still very limited [43,44], although some previous studies have already shown the effectiveness of LA against planktonic or sometimes attached *Campylobacter* cells [19,30,31].

Considering all the previous studies and to efficiently combat *Campylobacter* spp. in a sustainable way, this investigation sought to assess the inhibitory and eradicative efficacy of L-(+)-LA against a dual-species biofilm comprising two *C. jejuni* and one *C. coli* strain, all previously isolated from raw chicken meat. The influence of the abiotic substratum (polystyrene, PS; stainless steel, SS) on mixed-culture biofilm formation, without and in the presence of various LA sub-inhibitory concentrations, was also tested, together with the possible influence of aerobiosis conditions (microaerophilic, aerobic and CO_2_ enriched) on the resistance of the established biofilms to the acid. In all cases, LA’s biofilm inhibitory and destructive (disinfectant) actions were both determined against the mixed-strain consortium and each individual strain (that formed the mixed-culture consortium; no monoculture biofilms were tested). In addition, the planktonic populations of each strain grown under monoculture conditions were counted, together with those found in the vicinity of the 48 h mixed-culture biofilms (for each strain and in total), to check for any relationship between planktonic growth and biofilm-forming ability. Finally, the planktonic media of biofilm monocultures and mixed-culture biofilm were collected and subjected to an untargeted metabolomic analysis using liquid chromatography followed by tandem mass spectrometry (LC-MS/MS), to comparatively determine the main metabolites produced under each condition and thus to enrich our still limited knowledge on the physiology of that pathogen.

## 2. Materials and Methods

### 2.1. L(+)-Lactic Acid and Preparation of Its Stock Solution

L(+)-lactic acid (LA; 80% *v*/*v*, density: 1.2 g/mL) was purchased from PENTA Chemicals Unlimited (Prague, Czech Republic). For the preparation of its stock solution, it was dissolved in sterile distilled water (dH_2_O) at a concentration of 54.6% *v*/*v* (equal to 65.5% *w*/*v*). Once prepared, the solution was aseptically filtered through a microbiological filter (pore diameter 0.22 µm; Labbox Labware S.L., Barcelona, Spain) and then stored at 4 °C for up to one month.

### 2.2. Preparation of Sterile Chicken Juice (CJ)

Sterile CJ was prepared as previously described [44] and stored at −80 °C until its use as the nutrient substrate (supplement) for the growth of *Campylobacter* spp.

### 2.3. Bacterial Strains and Preparation of Their Working Cultures 

Three foodborne *Campylobacter* strains, consisting of two *C. jejuni* (CAMP_048 and CAMP_130) and one *C. coli* (CAMP_083), were used in this study, all previously isolated from raw chicken meat [45], along with the *C. jejuni* ATCC 33291 strain, a human outbreak isolate, as a reference. These four strains were selected to represent isolates with different features with regard to their biofilm-forming abilities, resistance to antibiotics, multidrug resistance (MDR) characteristics, colony morphotype, and rep-PCR genotypic patterns (see Table 1). All these inherent characteristics have been revealed in a preliminary screening of a large collection of foodborne *Campylobacter* spp. (data not presented). All strains were kept frozen at −80 °C in Mueller–Hinton (MH) broth (Oxoid Limited, Thermo Fisher Scientific Inc., Waltham, MA, USA) supplemented with 5% *v*/*v* laked horse blood (HB) (Thermo Fisher Scientific Inc.) and 20% *v*/*v* glycerol (Merck KGaA, Darmstadt, Germany), and their working cultures were prepared the days of the experiments as previously described [44].

### 2.4. Growth Dynamis of Campylobacter *spp.* Under Monoculture Planktonic Conditions

Bacteria from each final working culture (x4) were collected by centrifugation (3000× *g* for 10 min at 4 °C), washed once with quarter-strength Ringer’s solution (Lab M, Heywood, Lancashire, UK), and resuspended in MH broth supplemented with 5% *v*/*v* CJ (MH-CJ broth) to reach a cellular concentration of approximately 10^7^ CFU/mL. Bacteria from each of those suspensions were then used to inoculate (1:100), in duplicate, sterile MH-CJ broth in conical flasks (×4), which were statically incubated for 48 h at 42 °C under microaerophilic conditions (6.2–13.2% O_2_, 2.5–9.5% CO_2_; Oxoid CampyGen™ 2.5L Sachet; Thermo Fisher Scientific Inc.). During that incubation, one mL aliquots were collected from each culture at 0, 6, 24, and 48 h and used to enumerate viable bacteria (CFU/mL) through successive decimal dilutions (in quarter-strength Ringer’s solution) and agar plating onto MH-HB plates that were incubated at 42 °C for 48 h under microaerophilic conditions (before the enumeration of the colonies). 

### 2.5. Inhibitory Action of LA Against Mixed-Culture Campylobacter Biofilm Formation 

The three *Campylobacter* strains (CAMP_048, CAMP_083, and CAMP_130) were left to form together a dual-species biofilm on either PS surfaces (6-well PS microplates; 657102, Greiner Bio-One GmbH, Kremsmünster, Austria) or SS coupons (31 × 11 × 1 mm; type AISI 304) in the presence of four different LA concentrations (two-fold dilutions ranging from 4096 to 512 μg/mL; 0.34–0.043% *v*/*v*). For this, bacteria from each final working culture (in MH-CJ broth) were initially combined to achieve the same concentration for each strain (≈10^6^ CFU/mL). That mixed suspension was then used to inoculate (1:2) 2.5 mL of sterile MH-CJ broth also containing double the respective LA concentration (achieving a final volume of 5 mL). In the case of the PS biofilm, each inoculated broth was placed (in duplicate) in each well of the 6-well PS microplate, whereas in the case of the SS biofilm, that placement was carried out (again in duplicate) in a sterile glass test tube (1.6 cm diameter; 10 cm height) in which a sterile SS coupon had also been placed vertically. After placing the broth in the tube, the coupon was completely covered by it. For both abiotic surfaces, incubation took place statically for 48 h at 42 °C under microaerophilic conditions. In the case of the positive biofilm-forming control, the inoculated broth did not contain LA, whereas in the case of the negative control, the broth was not inoculated with bacteria. Following the 48 h of incubation, both the biofilm and surrounding planktonic bacteria were enumerated (for each strain and in total) by agar plating, as described in the following subsections (Section 2.5.1, Section 2.5.2 and Section 2.5.3), to determine the inhibitory action of LA against the sessile bacterial accumulation on the two surfaces (PS, SS), together with its accompanying effect on the concentration of the nearly adjacent free-swimming bacteria, respectively. 

#### 2.5.1. Enumeration of the Planktonic Bacteria

A one mL aliquot of each planktonic suspension was removed (in duplicate) from either the PS wells or the glass test tubes at the end of the 48 h incubation period, and transferred to a 1.5 mL Eppendorf^®^ tube, and was then mixed thoroughly using a vortexer (VXMNAL, Ohaus Europe GmbH, Nänikon, Switzerland). Subsequently, six serial decimal dilutions were prepared in a quarter-strength Ringer’s solution, and from each of those dilutions, 100 μL were spread (in duplicate) on MH-HB agar plates, which were incubated at 42 °C for 48 h under microaerophilic conditions. The developed colonies for each strain separately and in total were finally counted to determine the planktonic populations (CFU/mL) that existed in the wells/tubes at the time of sampling (48 h). It should be noted that the differentiation of each strain was easily achieved given their quite different macroscopic colony characteristics (Table 1).

#### 2.5.2. Detachment and Enumeration of the Biofilm Bacteria on PS

To quantify the biofilm cells that existed on the PS surface at the end of the 48 h incubation, the planktonic suspensions were totally removed, the wells were washed twice with quarter-strength Ringer’s solution (to remove the loosely attached cells), and 5 mL of this latter solution were added into each well. The submerged surface of each well was then thoroughly scratched with a plastic pipette tip, aiming to remove all the strongly attached and biofilm bacteria, which were again quantified (per strain and in total) by enumerating their discrete colonies on the MH-HB plates. The cellular concentrations of the biofilm-derived suspensions (CFU/mL) were finally converted to CFU/cm^2^, considering the total surface area (17 cm^2^) of each well that was initially covered by the 5 mL of the ΜH-CJ broth.

#### 2.5.3. Detachment and Enumeration of the Biofilm Bacteria on SS 

To quantify the biofilm cells that existed on the SS surface at the end of the 48 h incubation, the planktonic suspensions were again totally removed, and each SS coupon was washed twice with quarter-strength Ringer’s solution (to remove the loosely attached cells). It was then placed into a plastic 15 mL Falcon tube containing 5 mL of the same solution and 10 sterile glass beads (3 mm diameter; Witeg Labortechnik GmbH, Wertheim, Germany), and vortexed thoroughly for 2 min. The detached biofilm bacteria were again enumerated by agar plating as previously described, and the concentrations of sessile populations (CFU/cm^2^) were finally calculated (per strain and in total) considering the total surface area (0.84 cm^2^) of each fully immersed coupon. 

### 2.6. Eradicative Action of LA Against Mixed-Culture Campylobacter Biofilms 

Mixed-culture biofilms were again formed in MH-CJ for 48 h at 42 °C on either PS or SS, as previously described for the positive biofilm-forming control (Section 2.5), but this time, two aerobiosis biofilm-forming conditions were tested in parallel: either microaerophilic (6.2–13.2% O_2_, 2.5–9.5% CO_2_) (Oxoid CampyGen™ 2.5L Sachet; Thermo Fisher Scientific Inc.) or aerobic and CO_2_-enriched (15% O_2_, 3.5–9.0% CO_2_; Oxoid CO_2_Gen™ 2.5L Sachet; Thermo Fisher Scientific Inc.). To disinfect biofilm cells, following the removal of the planktonic and loosely attached cells, 5 mL of the respective LA solution (aquatic two-fold dilutions ranging from 65,536 to 4096 μg/mL; 5.46–0.34% *v*/*v*) were placed (in duplicate) in each PS well or glass test tube (containing the SS coupon). At all cases, disinfection took place for 15 min at room temperature. Following disinfection, each PS well or SS coupon was thoroughly washed with quarter-strength Ringer’s solution (to remove disinfectant residues) and the remaining viable biofilm bacteria (survivors) on each surface were detached and enumerated by plate counting on MH-HB agar (as previously described in Section 2.5.2 and Section 2.5.3). As a negative disinfection control, dH_2_O was used. Following the enumeration of the colonies, and the calculation of Log_10_ (CFU/cm^2^), log reductions were determined for each treatment as the difference between the log of survivors of the negative disinfection control and that of the respective LA treatment. 

### 2.7. Untargeted Metabolic Analysis of the Biofilm-Surrounding Planktonic Media

The three *Campylobacter* strains (CAMP_048, CAMP_083, and CAMP_130) were left to form biofilms on SS coupons, under either monoculture or mixed-culture conditions, by incubating them in MH-CJ broth at 42 °C for 48 h under microaerophilic conditions, as previously described for the positive biofilm-forming control (Section 2.5). Following incubation, 1 mL of each planktonic medium was aspirated from the glass test tube and centrifugated at 4000× *g* for 10 min to remove the bacteria (as a pellet). The supernatants were then collected and aseptically filtered through 0.22 µm microbiological filters (Labbox Labware S.L., Barcelona, Spain). These were finally stored at −80 °C until they were subjected to the subsequent process of metabolite extraction, which was carried out as previously described [48], with some slight modifications. In brief, approximately 400 μL of each supernatant were fully mixed with 800 μL of ice-cold methanol (MeOH) and left to stand at −20 °C for 30 min. To remove the precipitated proteins, the mixture was centrifuged at 16,000× *g* for 15 min. For each sample, 300 μL of the supernatant was dried in a SpeedVac at 45 °C. The obtained dry matter was reconstituted with 100 μL of MeOH:dH_2_O (50:50 *v*/*v*) and sonicated for 10 min. Finally, each sample was centrifuged at 16,000× *g* for 30 min and was then transferred to an LC vial. Metabolite extracts were analyzed using a Dionex UltiMate 3000 ultra-high-pressure liquid chromatography (UHPLC) system coupled with a heated electrospray ionization (HESI) source and a Q Exactive Focus (Thermo Fisher Scientific Inc.). For chromatographic separation, the reversed-phase Acquity UPLC HSS T3 C18 (2.1 × 100 mm, 1.8 μm) (Waters Corporation, Milford, MA, USA) was used. Mobile phase A consisted of water and 0.1% formic acid. Mobile phase B consisted of MeOH and 0.1% formic acid. The gradient was as follows: 0−1 min, 2% B; 1−6 min, 2−25% B; 6−10 min, 25–65% B; 10−15 min, 65−90% B; 15−21 min, 90–99.9% B; 21−23 min, 99.9% B; 23−24 min, 99.9–2% B; 24−3 min, 2% B. The constant flow rate was 0.3 mL/min and the column temperature was kept at 45 °C. The ion source was operated in positive and negative ionization modes and the various parameters were as follows: the spray voltage was +3.5 kV for both positive and negative mode, the capillary temperature was 350 °C, the heater temperature was 400 °C, the sheath gas flow was 45 arbitrary units, the auxiliary gas flow was 12 arbitrary units, the sweep gas flow was 0 arbitrary units, and the S-lens RF level was 50 V. Data acquisition was carried out by combining the full scan MS-AIF and the dd-MS2. For the full MS mode, the resolution was 70,000 and the m/z scan range was 67−1000. For the dd-MS2 mode, the resolution was 17,500 and the top 10 intense ions were fragmented by the stepped normalized collision energy at 10%, 30%, and 50%. Raw data were processed by Compound Discoverer 3.3 software (Thermo Fisher Scientific Inc.). The untargeted metabolomic workflow was used for peak alignment, peak detection, feature filtering and metabolite annotation. Online databases such as the Human Metabolomics database (HMDB; http://www.hmdb.ca/, accessed on 6 May 2024), MzCloud (https://www.mzcloud.org/, accessed on 6 May 2024) and ChemSpider (https://www.chemspider.com/, accessed on 6 May 2024) were used to tentatively determine the identities of significant metabolites. 

### 2.8. Statistics

Each experiment was repeated three times starting from independent bacterial cultures. Planktonic and biofilm plate counts (CFU/mL and CFU/cm^2^, respectively) were transformed to decimal logarithms before means and standard deviations were computed. The derived data on planktonic and biofilm logarithmic populations (log_10_ CFU/mL and log_10_ CFU/cm^2^, respectively), as well as those of log reductions, were then all submitted to analyses of variance (ANOVA), followed by Tukey’s multiple range post hoc honestly significant difference (HSD) tests for mean comparison, using the statistical software STATISTICA^®^ v12.0 (StatSoft Inc., Tulsa, OK, USA). Pearson correlation analysis was used to determine any correlation between biofilm counts on either PS or SS coupons (Log_10_ CFU/cm^2^) and surrounding planktonic populations (Log_10_ CFU/mL). Significant differences were always reported at a *p* level of <0.05. Regarding the statistical analysis of the metabolomic data, this was performed using Compound Discoverer 3.3 software. Differential metabolites with *p* < 0.001 and log_2_ FC > 3 were selected and screened for metabolite identification.

## 3. Results

### 3.1. Campylobacter Monoculture Planktonic Growth Dynamics 

**Figure 1:**
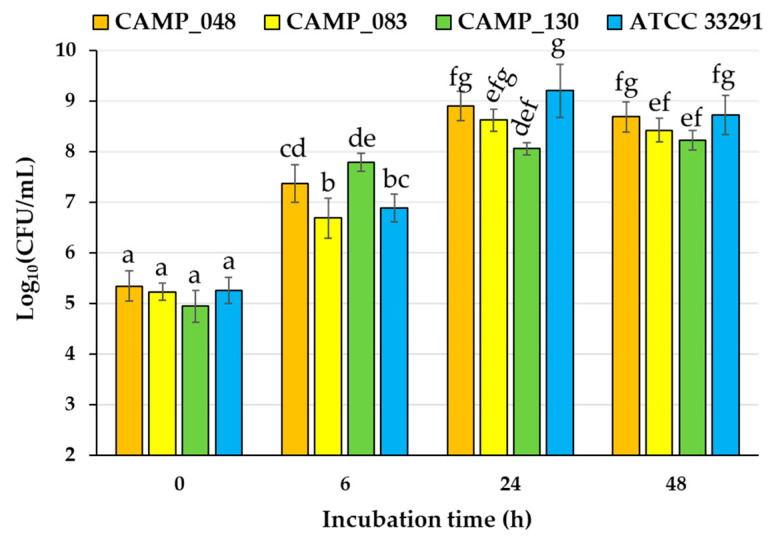
Logarithmic populations of planktonic cells (Log_10_ CFU/mL) for the three *C. jejuni* strains (CAMP_048, CAMP_130, and ATCC 33291) and the *C. coli* strain (CAMP_083). All strains were grown in MH-CJ broth at 42 °C for up to 48 h under microaerophilic conditions and viable bacteria were enumerated by agar plating at 0, 6, 24, and 48 h of incubation. Each bar represents the mean values ± standard deviations. The mean values followed by different superscript letters (a–g) differ significantly (*p* < 0.05).

The planktonic logarithmic populations (Log_10_ CFU/mL) for the three *C. jejuni* strains (CAMP_048, CAMP_130, and ATCC 33291) and the *C. coli* strain (CAMP_083), grown under monoculture microaerophilic conditions at 42 °C for up to 48 h, are shown in Figure 1 for the different time incubation intervals: 0, 6, 24, and 48 h.

Some significant variations in strain populations were observed at 6 and 24 h of incubation. However, no significant differences in the planktonic populations between the four strains existed at the end of incubation (48 h). Τhus, at this point, all strains exceeded 10^8^ CFU/mL in the population. 

### 3.2. Determination of LA’s Biofilm Inhibitory Action

**Figure 2:**
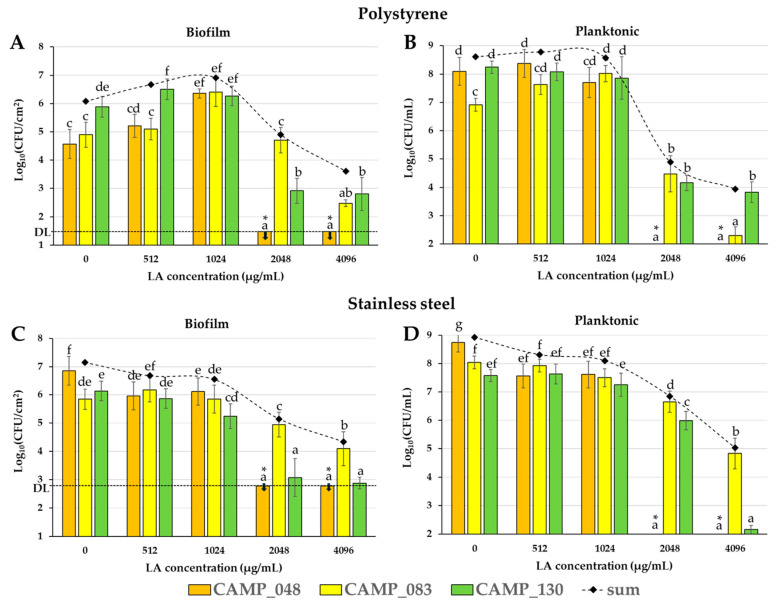
Biofilm and planktonic logarithmic populations (Log_10_ CFU/cm^2^ and Log_10_ CFU/mL, respectively) of each individual *Campylobacter* isolate (CAMP_048, CAMP_083, and CAMP_130) of the mixed-culture consortium, following the 48 h of incubation at 42 °C in MH-CJ broth, under microaerophilic conditions, and in the presence of the four tested LA concentrations (i.e., 512, 1024, 2048, and 4096 μg/mL; corresponding to the initial broth pH values of 5.41 ± 0.01, 4.47 ± 0.02, 3.87 ± 0.01, and 3.43 ± 0.03, respectively) for the two different biofilm formation experiments (PS and SS). (**A**). Biofilm populations on PS; (**B**). Planktonic populations in the surroundings of PS biofilms; (**C**). Biofilm populations on SS; (**D**). Planktonic populations in the surroundings of SS biofilms. The populations of the positive biofilm-forming control (without LA; 0 μg/mL) are also shown in each graph (initial broth pH value of 6.76 ± 0.02). Each bar denotes the mean values ± standard deviations. The total logarithmic populations (sum) for each treatment are shown as rhombuses connected by dotted curved lines. For clarity, standard deviation bars for those total population means were omitted. In each graph, the mean values followed by different superscript letters (a–f) differ significantly (*p* < 0.05). Population counts below the detection limits of the plate counting methods (1.47 Log_10_ CFU/cm^2^ for PS and 2.77 Log_10_ CFU/cm^2^ for SS, respectively) are denoted with asterisks (*) and inverted down arrows.

The biofilm and planktonic logarithmic populations (Log_10_ CFU/cm^2^ and Log_10_ CFU/mL, respectively), separately for each strain (CAMP_048, CAMP_083, and CAMP_130) and in total, upon growth in MH-CJ broth at 42 °C for 48 h under microaerophilic conditions in the presence of the four different LA concentrations (two-fold dilutions ranging from 4096 to 512 μg/mL), are shown in Figure 2, for the two different biofilm formation experiments (PS and SS).

**Figure 3:**
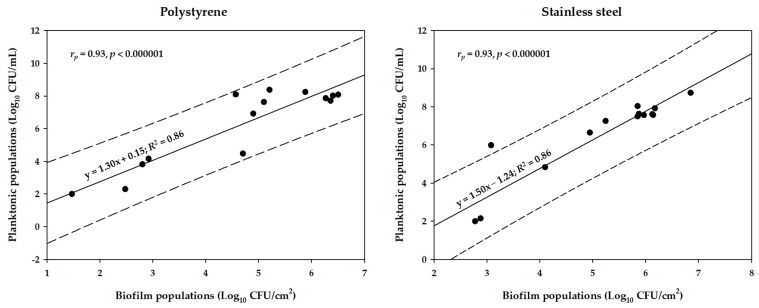
Correlation between biofilm populations on either PS or SS (Log_10_ CFU/cm^2^) and surrounding planktonic populations (Log_10_ CFU/mL) at the end of microaerophilic incubation (48 h). Solid lines illustrate linear regression equations, while dotted lines illustrate prediction intervals (*α* = 0.95). The Pearson’s correlation coefficients (*r_p_*), *p* values, mathematical equations of the regression plots, together with their regression coefficients (*R*^2^), are also shown. Dots represent mean values of all experiments (*n* = 15, three strains x five treatments, i.e., those shown in Figure 2). For greater clarity, the bars of standard deviations have been omitted.

A differential biofilm-forming behavior for each strain can be observed, which was also influenced by the abiotic substratum. Thus, when the incubation was carried out in pure MH-CJ broth (without the presence of LA), the *C. jejuni* CAMP_048 strain dominated on the SS surface (reaching a sessile population of 6.85 Log_10_ CFU/cm^2^) (Figure 2C), whereas, under the same conditions, the *C. jejuni* CAMP_130 was the one that dominated on the PS surface (reaching a sessile population of 5.88 Log_10_ CFU/cm^2^) (Figure 2A). This latter is quite interesting since the CAMP_130 strain had been previously found to be a weak biofilm-former on that surface when tested under monoculture conditions (Table 1)_,_ revealing the underlying influence of inter-strain interactions within the mixed-culture consortium. The incubation of the bacteria with increasing LA concentrations gradually decreased the mean numbers of biofilm and planktonic cells. Thus, when bacteria were incubated in the presence of 4096 μg/mL of LA (corresponding to a pH value of the MH-CJ broth of 3.43 ± 0.03), the mean biofilm populations were decreased by 2.47 and 2.82 logs on PS and SS, respectively (Figure 2A,C). At the same time, the populations of the surrounding planktonic cells were also decreased by 4.68 and 3.88 logs, respectively (Figure 2B,D). Therefore, a correlation seems to exist between the numbers of biofilm and planktonic cells for both experiments (PS, SS). This is indeed shown in Figure 3 by the linear regression plots for the two types of cells (biofilm, planktonic) for all three strains employed (CAMP_048, CAMP_083, and CAMP_130) and five treatments (concerning LA concentrations). The differential biofilm-forming and planktonic behaviors of each strain are more evident for the two highest LA concentrations that were tested (2048 and 4096 μg/mL), which were again dependent on the setup biofilm experiment (PS, SS). Thus, for instance, when the mixed-culture biofilm was left to be formed on PS and in the presence of 4096 μg/mL of LA, a sessile population of 2.80 Log_10_ CFU/cm^2^ was recovered for the *C. jejuni* CAMP_130 strain (Figure 2A). On the other hand, when the mixed-culture biofilm was developed on SS, again in the presence of that LA concentration, the latter strain could not be recovered (its sessile population was below the detection limit of the plate counting method; 2.77 Log_10_ CFU/cm^2^) and the biofilm population was in that case mainly composed of the *C. coli* CAMP_083 strain which managed to reach a sessile concentration of 4.10 Log_10_ CFU/cm^2^ (Figure 2C). The latter is again interesting given that the CAMP_083 strain presented the lowest number of sessile cells when biofilm formation was carried out in the absence of LA’s pressure. It thus seems that the intercellular interactions between the three strains not only exist but are also shaped by the respective environmental conditions (in this case, the type of abiotic substratum and LA’s presence). When looking at the biofilm and planktonic populations of the *C. jejuni* CAMP_048 strain, these were all below the plate count detection limits when LA was applied at the two highest tested concentrations (2048 and 4096 μg/mL). This probably denotes the increased sensitivity of this strain to LA and/or its reduced ability to antagonize the two other strains under those specific conditions, despite the fact that this strain dominated in the mixed-culture biofilm community formed on SS without the presence of LA (Figure 2C). The inter-strain interactions are indeed evident when looking at the planktonic populations of each strain in the case of the positive control (no LA exposure), which differed significantly (Figure 2B,D), although all three strains reached similar population amounts when grown under monoculture conditions (Figure 1). 

### 3.3. Determination of LA’s Biofilm Eradicative Action

**Figure 4:**
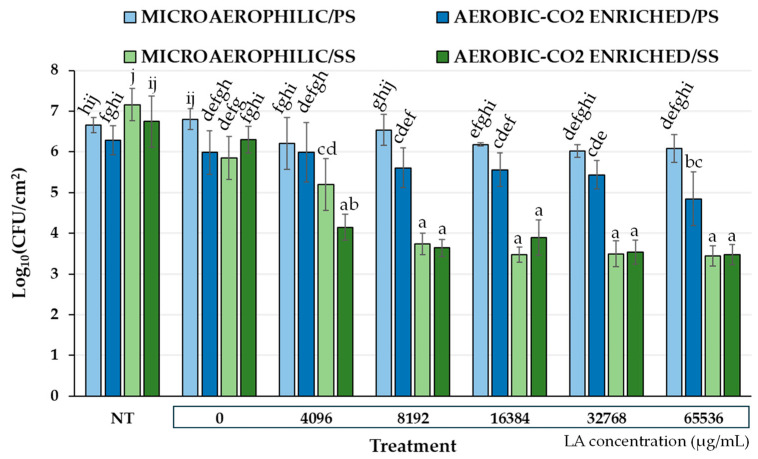
Total biofilm logarithmic populations (Log_10_ CFU/cm^2^) of the mixed-culture consortium following the 15 min LA exposure (disinfection). Prior to disinfection, biofilms had been left to be formed on either PS or SS surfaces incubated for 48 h at 42 °C in MH-CJ broth, under either microaerophilic or aerobic (and CO_2_-enriched) conditions. LA was tested in five different aquatic concentrations (i.e., 4096, 8192, 16,384, 32,768, and 65,536 μg/mL; corresponding to pH values of 2.91 ± 0.06, 2.73 ± 0.02, 2.49 ± 0.03, 2.29 ± 0.02, and 2.11 ± 0.02, respectively). The biofilm populations of the negative disinfection control (dH_2_O; 0 μg/mL LA; pH 7.17 ± 0.18) are also shown, together with those for the non-treated (NT) sample. Each bar denotes the mean values ± standard deviations. The mean values followed by different superscript letters (a–j) differ significantly (*p* < 0.05).

The total biofilm logarithmic populations (Log_10_ CFU/cm^2^) of the mixed-culture consortium survived on the two surfaces (PS, SS) following the 15 min LA exposure (disinfection) are presented in Figure 4, together with those for the negative disinfection control (dH_2_O; 0 μg/mL LA) and the non-treated (NT) sample. In the case of the NT sample, the values in the graphs denote the number of biofilm cells found on surfaces at the end of 48 h of incubation and just after the removal of planktonic and loosely attached cells (i.e., without any further treatment). Incubation under aerobic (and CO_2_-enriched) conditions seems to decrease biofilm formation on both surfaces; however, the differences were not significant (*p* > 0.05). However, in general, aerobiosis conditions seem to influence the resistance of *Campylobacter* spp. biofilms on PS, since the prior incubation under aerobic (and CO_2_-enriched) conditions resulted in fewer numbers of survivors. Nevertheless, this difference was more evident (significant) only when LA was applied at its maximum tested concentration (65,536 μg/mL) (*p* < 0.05). On the other hand, when the biofilms had been formed on SS, no significant differences in their resistance towards LA exposure were observed between the two different aerobiosis conditions, except for when LA was applied at 4096 μg/mL, where the biofilms that had been formed under aerobic conditions were again more sensitive to LA action. Although there were no significant differences in the numbers of the total biofilm cells between the two surfaces at the end of 48 h of incubation (these ranged from 6.28 to 7.16 Log_10_ CFU/cm^2^; NT), those biofilms that had been formed on SS presented significantly higher log reductions compared to the PS biofilms (*p* < 0.05). Thus, when LA was applied at its maximum tested concentration (65,536 μg/mL), there was no significant killing of biofilm cells that had been previously left to be accumulated on PS under the microaerophilic conditions, whereas there were recorded 2.41 and 2.83 log reductions for biofilm cells that had been accumulated on SS under microaerophilic and aerobic conditions, respectively. However, in all cases, the preformed mixed-culture biofilms could not be eradicated even when the acid was applied at concentrations exceeding 5% *v*/*v* (60,000 μg/mL), denoting their extremely high recalcitrance. 

**Figure 5:**
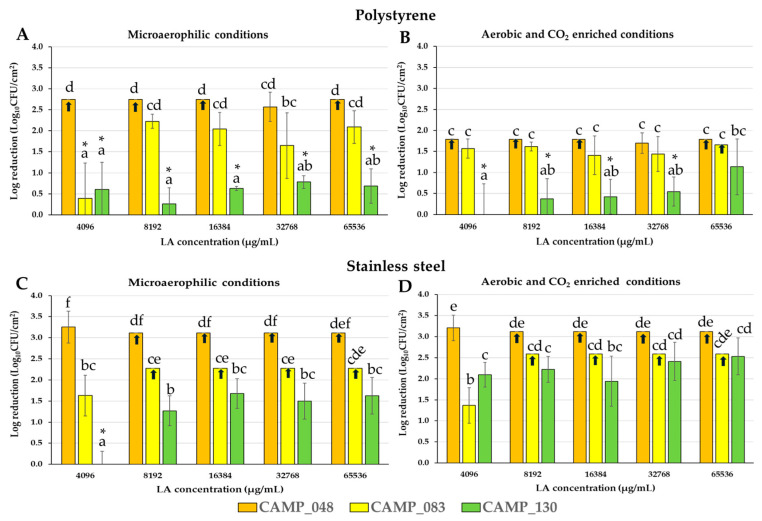
Log reductions for biofilm cells (Log_10_ CFU/cm^2^) separately for each strain (CAMP_048, CAMP_083, and CAMP_130) of the mixed-culture consortium following the 15 min LA exposure (disinfection). Prior to disinfection, biofilms had been left to be formed on either PS or SS surfaces incubated for 48 h at 42 °C in MH-CJ broth, under either microaerophilic or aerobic (and CO_2_-enriched) conditions. (**A**). Log reductions for biofilm cells that had been left to be formed on PS surfaces under microaerophilic conditions; (**B**). Log reductions for biofilm cells that had been left to be formed on PS surfaces under aerobic (and CO_2_-enriched) conditions; (**C**). Log reductions for biofilm cells that had been left to be formed on SS surfaces under microaerophilic conditions; (**D**). Log reductions for biofilm cells that had been left to be formed on SS surfaces under aerobic (and CO_2_-enriched) conditions. LA was tested in five different concentrations (i.e., 4096, 8192, 16,384, 32,768, and 65,536 μg/mL). Each bar denotes the mean values ± standard deviations. The mean values followed by different superscript letters (a–f) differ significantly (*p* < 0.05). Asterisks (*) denote log reductions that did not significantly differ from those of the negative disinfection control (dH_2_O). Τhe vertical arrows pointing upwards denote log reductions that were above the plate counting detection limits (depending on the surface and strain).

Figure 5 presents the log reductions for biofilm cells (Log_10_ CFU/cm^2^) separately for each strain (CAMP_048, CAMP_083, and CAMP_130) of the mixed-culture consortium following the 15 min LA exposure (disinfection). A differential behavior of each strain with regard to its LA sensitivity is again evident, that again seems to be influenced by the abiotic substratum (PS, SS) and biofilm-forming aerobiosis conditions (microaerophilic, aerobic). Thus, for instance, the biofilm cells of the CAMP_130 strain that had been previously left to be accumulated on surfaces under aerobic (and CO_2_-enriched) conditions, were reduced by 1.14 and 2.53 logs on PS and SS, respectively, when LA was applied at its maximum tested concentration (65,536 μg/mL) (Figure 5B,D). On the other hand, the log reductions that were recorded for the biofilm cells of that strain that had been previously left to be accumulated on surfaces under microaerophilic conditions were significantly lower and equal to 0.69 and 1.63 Log_10_ CFU/cm^2^ on PS and SS, respectively (Figure 5A,C). That specific *C. jejuni* strain presented the higher resistance to LA disinfection, since the log reductions that were recorded for it were in general significantly lower (*p* < 0.05) compared to those that were recorded for the two other strains (CAMP_048 and CAMP_083). On the contrary, the most sensitive strain to LA proved to be CAMP_048 (again *C. jejuni*). Thus, in almost all cases, this strain could not be recovered from the mixed-culture biofilm consortium following the application of the acid (its population was below the detection limits of the plate counting method), denoting perhaps its increased LA sensitivity. In agreement with that, the CAMP_048 strain also showed the greatest sensitivity to the acid in the biofilm-inhibition experiments (Figure 2). However, it is still worth noting that this strain had been previously found to be a strong biofilm-former on PS when tested under microaerophilic monoculture conditions (Table 1). 

### 3.4. Comparative Metabolomics of the Planktonic Media Between Campylobacter Monocultures and the Mixed Culture

**Figure 6:**
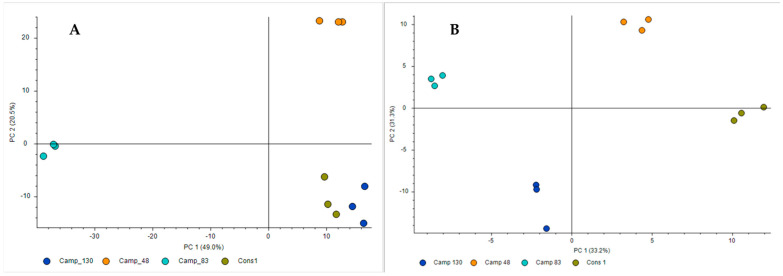
PCA score plots of LC-MS/MS-based metabolic profile from *Campylobacter* monocultures (CAMP_048, CAMP_083, and CAMP_130) and mixed-culture consortium (CONS1) in ESI+ (**A**) and ESI− (**B**) modes. The percentage on each axis represents the proportion of variance (R2X) explained by principal component one or two (PC1 or PC2).

LC-MS untargeted analysis revealed over 7500 features in all samples (3040 in positive ESI mode and 4500 in negative ESI mode). The 300 most dominant peaks were selected for further analysis. Putative identifications (IDs) of the features were determined using Compound Discoverer 3.3 software. Given the abundance of metabolites, we focused our analysis on the peaks that exhibited significant differences between the different samples, rather than attempting to identify all metabolites. Only results in which the similarity to the database reference compound was greater than or equal to 60 were used. A principal component analysis (PCA) model was then established in both positive and negative mode to evaluate any possible clustering of bacterial metabolite extracts of the three different *Campylobacter* strains (CAMP_048, CAMP_083, and CAMP_130) and their mixed-culture consortium (CONS1) without providing prior sample class information. Figure 6 illustrates the first two principal components (PCs), which collectively accounted for the majority of the observed variation. Thus, in the positive mode (Figure 6A), PC1 and PC2 represent 49.0% and 20.5% of the variation, respectively, while in the negative mode (Figure 6B), they represent 33.2% and 31.3% of the variation, respectively. It can be observed that in the PCA score plot of positive mode (Figure 6A), samples were clearly clustered into four clusters: CAMP_048, CAMP_083, CAMP_130, and CONS1. The distance was closer between CAMP_130 and CONS1 samples, meaning that the similarity of metabolite composition was higher. Conversely, the distances from these samples to CAMP_083 and CAMP_048 samples were further correspondingly, indicating that metabolic variations were gradually significant. In the PCA score plot of negative mode (Figure 6B), samples were also clearly clustered into four clusters: CAMP_048, CAMP_083, CAMP_130, and CONS1. In that case, the distance between all samples was far from each other, indicating that metabolic variations were gradually significant. Irrespective of the ESI mode, both graphs reveal a strain-specific metabolite production which might also be related to the strain-specific biofilm-forming and resistance behaviors which were previously noticed.

The Volcano plots demonstrating the levels of all the metabolites in the different pairs of samples (*p* < 0.001 and log_2_ FC > 3) in the two ESI- modes, together with Tables presenting those metabolites that showed a statistically significant difference between each sample pair, are also provided as Supplementary Materials (Figures S1–S12; Tables S1–S11).

## 4. Discussion

The ability of *Campylobacter* spp. to form and/or be included in biofilms on food processing surfaces, such as PS and SS, is strongly acknowledged to enhance their resistance to environmental stresses and disinfectants, complicating their eradication and increasing the likelihood of foodborne outbreaks [13]. Thus, efficient and sustainable strategies need to be applied within the food industry chain, starting from the poultry farm level, to inhibit sessile development by these bacteria and to eradicate any preformed sessile structure by them. Lactic acid was here found to inhibit dual-species *Campylobacter* biofilms by more than two logs (>99%) on PS and SS surfaces upon its application at 4096 μg/mL (equal to 0.34% *v*/*v*). These results suggest that LA could probably be effectively integrated into cleaning and sanitizing protocols in food processing environments to prevent biofilm formation and agree with the well-known efficacy of OAs as antimicrobial agents [22,23]. More specifically, LA has long been recognized for its antimicrobial properties and effectiveness against a wide range of foodborne pathogens also including *Campylobacter* spp. [19,20,29,30,31,32]. However, most of these older studies were occupied with planktonic or, in some cases, attached cells and did not deal with biofilm-enclosed microorganisms. The biofilm inhibition that was provoked by LA could be a direct result of the inhibition of cell proliferation (during their planktonic growth and/or their subsequent adhesion to surfaces) or could also be caused by any detrimental effects of the acid on the campylobacters’ biofilm-forming mechanisms (e.g., the blockage of cellular coaggregation, the interference with extracellular polysaccharide production, etc.). The strong correlation between the planktonic and biofilm cell numbers that was here observed surely advocates towards the first scenario, without, however, being possible to exclude any other in parallel biofilm-specific responses. It is indeed known that the planktonic growth rate of a given microorganism is not always related to its biofilm-forming capacity, while there are also special cases of biofilm formation in environments where microbial cells still fail to multiply planktonically [49,50]. In addition, differences in biofilm formation were observed between the two tested surfaces. The implications of these surface-dependent differences in biofilm development are crucial for food processing industries because they can significantly impact the ability of microorganisms to persist, resist disinfection, and contribute to food contamination. Effective biofilm control strategies, such as the use of OAs, thus need to be tailored to account for these variations in biofilm resilience depending on the surface and environmental conditions.

It is worth noting that the MICs of LA against the three strains here studied (CAMP_048, CAMP_083, and CAMP_130), upon their planktonic growth in MH broth, with or without 5% *v*/*v* HB, at 42 ◦C for 48 h under microaerophilic conditions, had been previously found to vary from 1024 to 2048 μg/mL (equal to 0.09–0.17% *v*/*v*), depending on the isolate and blood presence or not [44]. In that same older study, LA needed to be applied at either 1024 or 2048 μg/mL to completely inhibit biofilm formation by CAMP_048 and CAMP_083/CAMP_130 strains, respectively, on 96-well PS microtiter plates, upon growth under monoculture conditions in MH-CJ broth at 42 ◦C for 48 h under microaerophilic conditions (i.e., the same environmental conditions as those tested here, with respect to the abiotic substratum, growth medium, temperature, time, and atmosphere). Indeed, that increased acid sensitivity of the *C. jejuni* CAMP_048 strain was also evident in the mixed-culture conditions of the present study, where its biofilm populations on both surfaces (PS, SS) were always below the plate count detection limits when LA was applied, as the inhibitor, at the two highest tested concentrations (1024 and 2048 μg/mL), whereas in almost all cases of disinfection treatments, this strain presented the highest log reductions and was not recovered. Interestingly enough, however, the biofilm populations of that strain surpassed 10^6^ CFU/cm^2^ when LA was applied during the course of biofilm formation at 1024 μg/mL, that is at the concentration that had been previously shown to totally inhibit biofilm development by that strain under monoculture conditions. It is thus clear that the inter-strain interactions encountered within and upon the development of the mixed-culture consortium can significantly increase the resilience of each biofilm member and in total against LA action. Those determining effects of intra- and inter-species interactions on biofilm formation and the subsequent recalcitrance of the enclosed sessile cells are indeed well-documented for several important foodborne bacterial pathogens [18] and guided us to test in this study LA action against a carefully selected mixed-culture *Campylobacter* consortium. 

Thus, when multiple microbial strains and/or species coexist in a biofilm, they often form complex, synergistic relationships that enhance the biofilm’s structural integrity and resilience. Several factors may contribute to this phenomenon. First, intercellular signaling through quorum sensing (QS) can regulate biofilm development, enhancing the production of extracellular polymeric substances (EPSs) that form a protective matrix around the cells. This matrix acts as a physical barrier, preventing some antimicrobials from penetrating deep into the biofilm and reaching the embedded cells. Additionally, different strains and/or species may occupy specific niches within the biofilm, with some strains/species being more adept at surviving external stresses or metabolizing antimicrobial agents, indirectly protecting other strains/species. For instance, one species may metabolize LA or alter the local microenvironment (e.g., pH, oxygen levels) in ways that reduce LA’s effectiveness against the biofilm as a whole. Moreover, intercellular competition and cooperation may influence biofilm architecture, leading to a denser, more heterogeneous structure that further enhances resistance. Strains and/or species with stronger biofilm-forming capabilities can reinforce the biofilm’s foundation, while weaker strains/species may benefit from the shared protective environment. This structural complexity, combined with metabolic diversity, makes it harder for antimicrobials like LA to act uniformly across the biofilm. To effectively counteract this enhanced resistance, treatments must address both the physical barriers created by intercellular interactions and the metabolic adaptations that contribute to biofilm resilience. Indeed, strategies such as combining LA with enzymes that degrade EPSs, or using agents that disrupt QS, may help weaken the biofilm’s defenses and improve eradication outcomes. Understanding the specific intercellular dynamics at play in mixed-culture biofilms is therefore essential for designing more effective antimicrobial strategies. It should be noted, however, that our research still cannot fully imitate real-world conditions and should be further expanded to include a wider range of *Campylobacter* strains and other microbial species that could be potentially encountered within the food industry, together with *Campylobacter* spp. (e.g., *Pseudomonas*, *Acinetobacter*, *Psychrobacter*, etc.) [51], as well as trying to form such mixed-species biofilms under other environmental conditions mimicking those within food processing (mainly concerning temperature, nutrients, the presence of organic matter, and oxygen concentrations). 

Current results also highlight the great resilience of the established biofilms, which could not be eradicated even at LA concentrations exceeding 5% *v*/*v*. Undoubtedly, this finding underscores the significant protective nature of biofilms, particularly their ability to shield bacterial cells from antimicrobial agents, thus complicating eradication efforts [9]. In addition, the differential responses of biofilm cells based on the type of surface (PS vs. SS) and the biofilm-forming aerobiosis conditions (microaerophilic vs. aerobic) further suggest that the efficacy of LA can be significantly influenced by environmental factors, which could be crucial for tailoring disinfection strategies in industrial settings. Not surprisingly, some other previous studies have shown how varying oxygen levels can significantly influence the effectiveness of antimicrobial agents, highlighting the importance of considering oxygen availability in both clinical and industrial settings [52,53,54,55,56]. In addition, previous research has demonstrated varying effects of OAs on *Campylobacter* spp., often dependent on the type of acid and its concentration. In such a recent study, Bai et al. successfully developed a disinfectant consisting of two OAs, citric acid (CA) and LA, and sodium dodecyl sulfate (SDS) that presented a synergistic bactericidal effect against drug-resistant *Campylobacter* strains [29]. The bactericidal efficacy of the disinfectant improved with increased concentrations of the three active ingredients, while LA needed to be applied at 0.025% *w*/*w*, at least, to reach a 100% reduction in *Campylobacter* during a 15 s in vitro treatment. In addition, the OA compound disinfectant (CA-0.06% *w*/*w*, LA-0.08% *w*/*w*, SDS-0.02% *w*/*w*) showed an excellent bactericidal effect during an on-site 15 min disinfection in chicken slaughterhouses by significantly reducing the number of pathogenic *Campylobacter* spp. on chicken carcasses and processing tools (such as knives and gloves). In another study, Beier et al. determined the MICs of six OAs (including LA) against 96 *C. jejuni* isolates from broiler chicken houses [19]. They found that the effectiveness of OAs varied and correlated with the concentrations of dissociated OAs rather than with the pH levels or the concentrations of undissociated molecules. These authors recommended a minimum concentration of dissociated LA of 40 mM (equal to 0.3% *v*/*v* or 3603.2 μg/mL) in a carcass wash to potentially remove or inhibit 97% or more of the *C. jejuni* bacteria. However, it is now clear from the results of the present study that such a concentration seems to be too low and will likely prove totally insufficient if *Campylobacter* spp. have previously managed to form a biofilm on the surface to be disinfected.

In another relevant recent study, Carvalho et al. investigated the antibiofilm activity of a biosurfactant (rhamnolipid) and two OAs (malic acid and CA) against some common foodborne pathogens, also including *C. jejuni*, under various conditions relevant to real-world poultry processing environments [43]. All three compounds exhibited antibiofilm activity under various temperatures (4, 12, and 25 °C), compound concentrations, and contact times (5 and 10 min), with higher temperatures and concentrations being generally found to improve the antibiofilm efficacy. In addition, the effectiveness of the treatments varied among the tested pathogens, highlighting the need for targeted approaches depending on the specific pathogen and environmental conditions within food processing settings. Like in the present study, in that older study also, the three compounds were tested for both preventing the formation of biofilms and removing established biofilms. While effective in both roles, there were variations in their performance, also in agreement with the results of the current study, suggesting that optimal formulations and application strategies might differ based on whether the goal is biofilm prevention or removal. Another pertinent study evaluated the efficacy of an LA wash and modified atmosphere packaging (MAP) on reducing *C. jejuni* counts on chicken legs during refrigerated storage [30]. Washing chicken legs with a 2% LA solution significantly reduced *C. jejuni* counts by about 1.5 log units immediately after treatment compared to controls washed with dH_2_O. Interestingly, the combination of an LA wash with MAP (40% CO_2_/60% N_2_) further reduced pathogenic counts across various storage days and conditions, emphasizing the potential of combined treatments for enhancing food safety. 

Indeed, the integration of LA with other antimicrobial agents and/or physical methods might significantly enhance its efficacy and thus achieve more comprehensive biofilm control and eradication. This sounds quite important when one also considers the inadequate efficacy of LA against the established *Campylobacter* biofilms when applied alone, which was noticed in the current study. For instance, strong synergistic effects upon the combination of several OAs against *Campylobacter* spp. have been previously described [20]. In another study, the combination of LA and reuterin (a neutral broad-spectrum antimicrobial compound produced from glycerol by *Lactobacillus reuteri*) showed a synergistic effect, significantly reducing *Salmonella enterica* counts on chicken carcasses compared to when they were used alone [57]. Thus, expanding this type of research to assess the synergy of LA with other antimicrobials or mechanical interventions could be vital for overcoming the recalcitrance of biofilms in food processing environments. Such combined strategies may indeed offer significant potential to confront biofilms in those and other environments. For instance, by integrating LA with other antimicrobial agents, such as OAs or surfactants like SDS, or with physical treatments like heat, ultrasonic treatments, high-pressure washing, or MAP, or with biological agents such as enzymes, the overall effectiveness could be enhanced. These synergistic approaches could indeed improve antimicrobial penetration, disrupt the biofilm structure, and increase microbial susceptibility. Undoubtedly, this multi-targeted approach seems essential to effectively eradicate established biofilms, especially multispecies ones, which are highly resistant to single treatments, thus ensuring better biofilm control and food safety. 

Although LA is already used within food industries (ingredient E270), there are not many data comparing its effectiveness to that of other antimicrobials. In a previous relevant study of our group, the antibacterial and antibiofilm actions of LA were determined and compared to those of a general-purpose biocide and cationic surfactant (benzalkonium chloride; BAC), and a widely used macrolide antibiotic (erythromycin; ERY), against 12 *Campylobacter* spp. raw chicken meat isolates [44]. In that study, the MICs and MBICs of LA varied from 1024 to 2048 μg/mL and were almost always higher compared to those recorded for BAC and ERY, thus revealing its reduced efficiency compared to the other two compounds. On the other hand, Bautista et al. evaluated the bactericidal effects of LA, chlorine, trisodium phosphate (TSP), and a commercial phosphate blend (Avgard^TM^) on turkey carcasses. Lactic acid at 4.25% *w*/*w* was found to be the most effective, reducing microbial load and coliforms by more than 95% and also having some significant effects on *Salmonella* spp., whereas chlorine and TSP were not effective against that pathogen [58]. In another study, the antimicrobial activity of 3-phenyllactic acid (PLA) produced by LAB was compared with LA and phenolic acids such as gallic, caffeic, and ferulic acids. PLA showed lower MIC values against *Listeria innocua* than LA and the phenolic acids, indicating a stronger antimicrobial effect [59]. Oh and Marshall assessed the antimicrobial activity of LA, ethanol, and glycerol monolaurate (monolaurin; a dietary supplement and food additive that is used as an emulsifier or preservative) against *Listeria monocytogenes*. Lactic acid showed an MIC value of 0.5% (5000 μg/mL), which decreased when combined with ethanol, indicating some level of interaction [60]. MIC values of monolaurin and ethanol alone were 10 μg/mL (0.001%) and 50 000 μg/mL (5%), respectively. In another study, the effects of LA (2 or 4% *w*/*v*) were compared with peroxyacetic acid (0.02% *w*/*v*) and acidified sodium chlorite (0.16% *w*/*v*) on chilled beef carcasses [61]. Lactic acid at 4% *w/v* was more effective in reducing bacterial numbers compared to the two other solutions. These previous studies confirm the versatility and effectiveness of LA as an antimicrobial agent, often outperforming or enhancing the effects of other antimicrobial compounds. 

Although it is rather expected, another notable aspect of the current research that should be referenced is the strain-specific biofilm-behaviors and responses to LA exposure. Thus, the three tested *Campylobacter* strains were shown to exhibit different biofilm-forming abilities and varying levels of resistance, also depending on the abiotic substratum (PS, SS) and aerobiosis conditions (microaerophilic, aerobic). As previously mentioned, this variability underscores the need for targeted approaches in biofilm control strategies that will consider the specific strains present, in situ environmental conditions, and intercellular interactions within the biofilm. In addition, this strain variability of the behavior of foodborne bacterial pathogens has been previously described [62,63] and seems to be following the results of the metabolic analysis, which was here additionally performed. Hence, following the latter analysis, a distinct clustering of the different samples (strains) was evident. Indeed, such a metabolomic profiling could prove to be a valuable tool in elucidating the complex biochemical pathways that may contribute to biofilm formation and persistence [48]. Towards that end, other previous metabolomics studies on *Campylobacter* spp., particularly *C. jejuni*, have already identified a diverse array of metabolites that reflect its unique metabolic adaptations required for colonization and resistance [64,65,66]. Metabolic diversity between bacterial strains seems to play a crucial role in their ability to form biofilms and resist antimicrobial agents like LA. Each strain can produce different metabolites, which may influence biofilm formation mechanisms, such as cell adhesion and extracellular matrix production. These metabolic differences can thus lead to varying levels of biofilm robustness and resilience. Strains with unique metabolic adaptations may enhance their survival within biofilms, particularly by better managing stress and nutrient conditions. Moreover, this metabolic variability can affect how different strains respond to LA. Some strains may metabolically adjust to acidic environments more effectively, leading to greater resistance, while others are more vulnerable to the acid’s inhibitory effects. In mixed-species biofilms, metabolic interactions between strains can further enhance their collective resistance to antimicrobials, such as LA.

Although the purpose of the present study was not to go deeply into the specific metabolites produced in each case, the use of untargeted metabolomics that was chosen here allows for a comprehensive analysis of the metabolites produced by each strain under monoculture conditions, also in comparison to those produced under mixed-culture conditions, and such an approach might hopefully reveal potential targets for future interventions. Thus, future studies could expand on this preliminary work by employing targeted metabolomics, transcriptomics, proteomics, or even genomic approaches to identify specific metabolic pathways that are critical for biofilm formation and maintenance, together with those required for gut colonization and the evasion of host defenses [67,68,69,70]. Such knowledge could ultimately lead to the development of new interventions that disrupt these pathways, thereby enhancing the efficacy of biofilm control measures. 

In addition, further studies are recommended to investigate the specific mechanisms through which LA inhibits and disrupts biofilm formation, together with the mechanisms underlying the strain-specific differences in LA sensitivity. Understanding these mechanisms will aid in optimizing the use of LA and potentially other OAs in targeted antimicrobial strategies. As previously mentioned, it sounds also quite useful to expand this research to include a wider range of *Campylobacter* strains and other microbial species and to test LA in real-world food processing settings. Such studies could provide valuable data on LA’s practical effectiveness and operational feasibility and validate its generalizability as a biofilm inhibitor. Last but not least, investigating the long-term impacts of regular LA use on microbial communities within food processing environments is crucial. This includes assessing potential shifts in microbial ecology, resistance development, and the overall sustainability of LA use.

## 5. Conclusions

L(+)-lactic acid (LA) was found to effectively inhibit the formation of dual-species biofilms of *C. jejuni* and *C. coli* on both PS and SS surfaces. At higher concentrations (up to 0.34% *v*/*v*), LA reduced biofilm formation by more than 2 logs (>99%) on these surfaces, indicating its potential as a preventive measure in food processing environments. However, despite its effectiveness in inhibiting biofilm formation, LA was not able to completely eradicate the established biofilms, even when applied at concentrations exceeding 5% *v*/*v*, highlighting the great resilience of *Campylobacter* biofilms, particularly those formed in mixed-culture environments. The effectiveness of LA in inhibiting and eradicating biofilms was influenced by the type of surface (PS or SS) and the environmental conditions (microaerophilic or aerobic with CO_2_ enrichment). Biofilms formed on SS were more susceptible to LA disinfection than those on PS, particularly under aerobic conditions. In addition, this study demonstrated that different *Campylobacter* strains exhibited different biofilm-forming abilities and varying levels of resistance to LA, with some strains being more resilient within biofilms than others, while those strain-specific behaviors were further influenced by the environmental conditions (surface type and aerobiosis conditions) and inter-strain interactions. In agreement with the previous studies mentioned, untargeted metabolomic analysis revealed distinct metabolic profiles for each *Campylobacter* strain and their mixed-culture consortium, suggesting that some metabolic differences may contribute to the differential phenotypes observed. Some specific recommendations for food industry professionals that arise from our study’s results are to use LA at concentrations of at least 0.34% *v*/*v* to prevent *Campylobacter* biofilm formation on surfaces like SS and PS. For established biofilms, LA should, however, be used at much higher concentrations (>5% *v*/*v*) and combined with other antimicrobials or mechanical cleaning for better efficacy. In addition, disinfection methods need to be adjusted based on specific environmental conditions and *Campylobacter* strains present in the facility to enhance their efficiency. Overall, it can be concluded that while LA shows promise as a green antimicrobial agent for inhibiting *Campylobacter* biofilm formation, additional measures are needed to eradicate established biofilms effectively, particularly in mixed-culture environments. Such an effective and global biofilm management strategy within the food industry chain could ensure food safety and protect public health.

## Figures and Tables

**Table 1:** *Campylobacter* strains used in this study and their relative information.

Strain Code	Species	Biofilm-Forming Ability ^1^		MIC LA (μg/mL) ^2^	MBIC LA (μg/mL) ^3^	Antibiotic Resistance ^4^	MDR ^5^	Colony Morphotype ^6^	Rep-PCR Group ^7^
		Microaerophilic	Aerobic-CO_2_ Enriched						
CAMP_048	*C. jejuni*	strong	moderate	2048	1024	SXT (25 μg), CTX (5 μg), CAZ (10 μg)	-	small translucent	a
CAMP_083	*C. coli*	weak	moderate	2048	2048	SXT (25 μg), CTX (5 μg), CAZ (10 μg)	-	white	c
CAMP_130	*C. jejuni*	weak	moderate	2048	2048	TE (30 μg), CIP (5 μg), CAZ (10 μg)	+	gray spread	c
ATCC 33291	*C. jejuni*	zero	zero	ND ^8^	ND ^8^	-	-	small translucent	b

^1^ The biofilm-forming ability of each strain was categorized based on the crystal violet (CV) staining method for biofilm quantification in 96-well PS microtiter plates proposed by Stepanovic et al. [46]. For this, biofilms were left to be formed statically for 48 h at 42 °C in Mueller–Hinton (MH) broth supplemented with 5% *v*/*v* CJ (MH-CJ broth), under either microaerophilic (6.2–13.2% O_2_, 2.5–9.5% CO_2_) (Oxoid CampyGen™ 2.5L Sachet; Thermo Fisher Scientific Inc.) or aerobic-CO_2_-enriched (15% O_2_, 3.5–9.0% CO_2_) conditions (Oxoid CO_2_Gen™ 2.5L Sachet; Thermo Fisher Scientific Inc.). ^2^ The Minimum Inhibitory Concentration (MIC) of LA against each strain was determined through the broth microdilution method by culturing strains for 48 h at 42 °C in Mueller–Hinton (MH) broth supplemented with 5% *v*/*v* laked horse blood (MH-HB broth) under microaerophilic conditions (6.2–13.2% O_2_, 2.5–9.5% CO_2_) (Oxoid CampyGen™ 2.5L Sachet; Thermo Fisher Scientific Inc.) [44]. ^3^ The Minimum Biofilm Inhibitory Concentration (MBIC) of LA against each strain was determined through biofilm inhibition CV assay on 96-well PS microtiter plates by culturing strains for 48 h at 42 °C in MH-CJ broth under microaerophilic conditions (6.2–13.2% O_2_, 2.5–9.5% CO_2_) (Oxoid CampyGen™ 2.5L Sachet; Thermo Fisher Scientific Inc.) [44]. ^4^ The resistance of the bacteria to antibiotics was tested through the disk diffusion susceptibility test (Kirby–Bauer method) and categorized based on the clinical breakpoints v14.0 published by the European Committee on Antimicrobial Susceptibility Testing (EUCAST) [47]. ^5^ Multidrug resistance (MDR) was defined as the resistance of a given strain to antibiotics of at least three different classes based on the EUCAST clinical breakpoints [SXT: Trimethoprim-sulfamethoxazole 1:19 (miscellaneous agents); CTX: Cefotaxime (cephalosporins); CAZ: Ceftazidime (cephalosporins); TE: Tetracycline (tetracyclines); CIP: Ciprofloxacin (floroquinolones)]. ^6^ Following growth for 48 h at 42 °C on MH agar supplemented with 5% *v*/*v* laked horse blood (HB) under microaerophilic conditions. ^7^ The clustering of the strains was performed following rep-PCR-based typing with the GTG_5_ primer [44]. ^8^ Not determined.

## Data Availability

The data presented in this study are contained within the article or Supplementary Materials.

## Supplementary Materials

The following supporting information can be downloaded at: https://www.mdpi.com/article/10.3390/microorganisms12112124/s1, Figure S1: Volcano plot demonstrating the levels of all the metabolites in the Camp_130 vs. Camp_48 (*p* < 0.001 and log_2_ FC > 3) in ESI+ mode. In the colored areas, the metabolites that show a statistically significant difference are depicted; Figure S2: Volcano plot demonstrating the levels of all the metabolites in the Camp_130 vs. Camp_83 (*p* < 0.001 and log_2_ FC > 3) in ESI+ mode. In the colored areas, the metabolites that show a statistically significant difference are depicted; Figure S3: Volcano plot demonstrating the levels of all the metabolites in the Camp_130 vs. Cons1 (*p* < 0.001 and log_2_ FC > 3) in ESI+ mode. In the colored areas, the metabolites that show a statistically significant difference are depicted; Figure S4: Volcano plot demonstrating the levels of all the metabolites in the Camp_48 vs. Camp83 (*p* < 0.001 and log_2_ FC > 3) in ESI+ mode. In the colored areas, the metabolites that show a statistically significant difference are depicted; Figure S5: Volcano plot demonstrating the levels of all the metabolites in the Camp_48 vs. Cons1 (*p* < 0.001 and log_2_ FC > 3) in ESI+ mode. In the colored areas, the metabolites that show a statistically significant difference are depicted; Figure S6: Volcano plot demonstrating the levels of all the metabolites in the Camp_83 vs. Cons1 (*p* < 0.001 and log_2_ FC > 3) in ESI+ mode. In the colored areas, the metabolites that show a statistically significant difference are depicted; Figure S7: Volcano plot demonstrating the levels of all the metabolites in the Camp_130 vs. Camp_48 (*p* < 0.001 and log2 FC > 3) in ESI- mode. In the colored areas, the metabolites that show a statistically significant difference are depicted; Figure S8: Volcano plot demonstrating the levels of all the metabolites in the Camp_130 vs. Camp_83 (*p* < 0.001 and log_2_ FC > 3) in ESI- mode. In the colored areas, the metabolites that show a statistically significant difference are depicted; Figure S9: Volcano plot demonstrating the levels of all the metabolites in the Camp_130 vs. Cons1 (*p* < 0.001 and log2 FC > 3) in ESI- mode. In the colored areas, the metabolites that show a statistically significant difference are depicted; Figure S10: Volcano plot demonstrating the levels of all the metabolites in the Camp_48 vs. Camp83 (*p* < 0.001 and log2 FC > 3) in ESI- mode. In the colored areas, the metabolites that show a statistically significant difference are depicted; Figure S11: Volcano plot demonstrating the levels of all the metabolites in the Camp_48 vs. Cons1 (*p* < 0.001 and log2 FC > 3) in ESI- mode. In the colored areas, the metabolites that show a statistically significant difference are depicted; Figure S12: Volcano plot demonstrating the levels of all the metabolites in the Camp_83 vs. Cons1 (*p* < 0.001 and log2 FC > 3) in ESI- mode. In the colored areas, the metabolites that show a statistically significant difference are depicted; Table S1: LC/MS upregulated features that differed significantly between Camp_130 and Camp_48 in ESI+ and ESI- mode. Data represents directed effected size for each bacteria obtained from volcano plot and significant at *p*-value ≤ 0.001; Table S2: LC/MS downregulated features that differed significantly between Camp_130 and Camp_48 in ESI+ and ESI- mode. Data represents directed effected size for each bacteria obtained from volcano plot and significant at *p*-value ≤ 0.001; Table S3: LC/MS upregulated features that differed significantly between Camp_130 and Camp_83 in ESI+ and ESI- mode. Data represents directed effected size for each bacteria obtained from volcano plot and significant at *p*-value ≤ 0.001; Table S4: LC/MS downregulated features that differed significantly between Camp_130 and Camp_83 in ESI+ and ESI- mode. Data represents directed effected size for each bacteria obtained from volcano plot and significant at *p*-value ≤ 0.001; Table S5: LC/MS downregulated features that differed significantly between Camp_130 and Cons1 in ESI+ and ESI- mode. Data represents directed effected size for each bacteria obtained from volcano plot and significant at *p*-value ≤ 0.001; Table S6: LC/MS upregulated features that differed significantly between Camp_48 and Camp_83 in ESI+ and ESI- mode. Data represents directed effected size for each bacteria obtained from volcano plot and significant at *p*-value ≤ 0.001; Table S7: LC/MS downregulated features that differed significantly between Camp_48 and Camp_83 in ESI+ and ESI- mode. Data represents directed effected size for each bacteria obtained from volcano plot and significant at *p*-value ≤ 0.001; Table S8: LC/MS upregulated features that differed significantly between Camp_48 and Cons1 in ESI+ and ESI- mode. Data represents directed effected size for each bacteria obtained from volcano plot and significant at *p*-value ≤ 0.001; Table S9: LC/MS downregulated features that differed significantly between Camp_48 and Cons1 in ESI+ and ESI- mode. Data represents directed effected size for each bacteria obtained from volcano plot and significant at *p*-value ≤ 0.001; Table S10: LC/MS upregulated features that differed significantly between Camp_83 and Cons1 in ESI+ and ESI- mode. Data represents directed effected size for each bacteria obtained from volcano plot and significant at *p*-value ≤ 0.001; Table S11: LC/MS downregulated features that differed significantly between Camp_83 and Cons1 in ESI+ and ESI- mode. Data represents directed effected size for each bacteria obtained from volcano plot and significant at *p*-value ≤ 0.001.
