# Supplementary material for: Investigating the Potential of L(+)-Lactic Acid as a Green Inhibitor and Eradicator of a Dual-Species Campylobacter spp. Biofilm Formed on Food Processing Model Surfaces
Source: Microorganisms. 2024 Oct 23;12(11):2124. doi: 10.3390/microorganisms12112124 (PMC11596057; doi:10.3390/microorganisms12112124)
Supplement: Supplementary file 1 [file microorganisms-12-02124-s001.zip › microorganisms-3232463-supplementary.pdf]

SUPPLEMENTARY MATERIAL

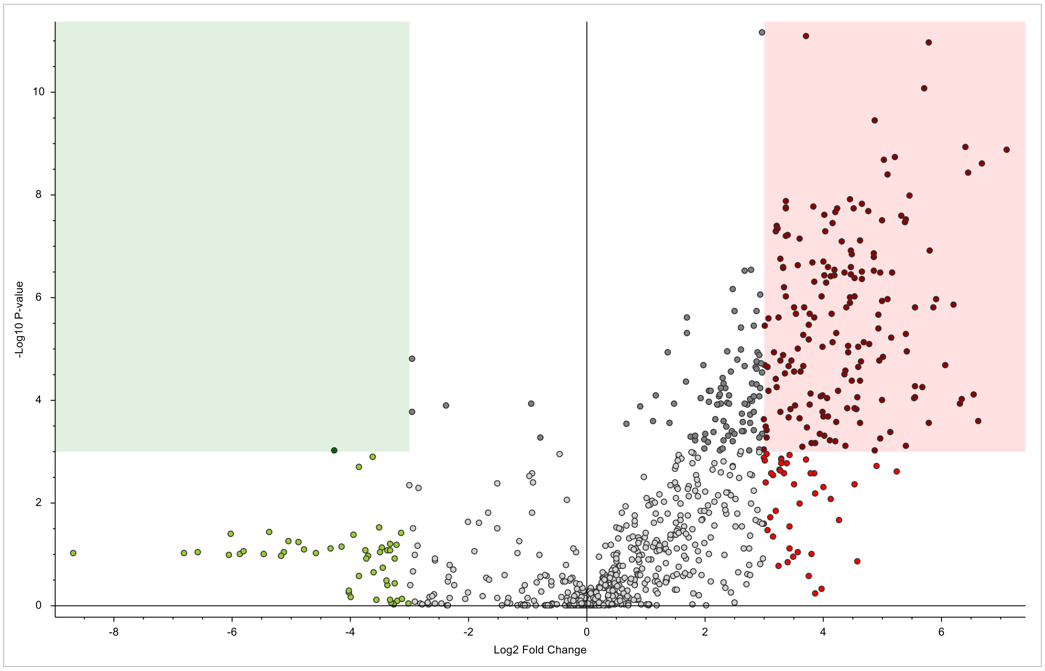

Figure S1. Volcano plot demonstrating the levels of all the metabolites in the Camp_130 vs. Camp_48 (p<0.001 and log2 FC>3) in ESI+ mode. In the colored areas, the metabolites that show a statistically significant difference are depicted.

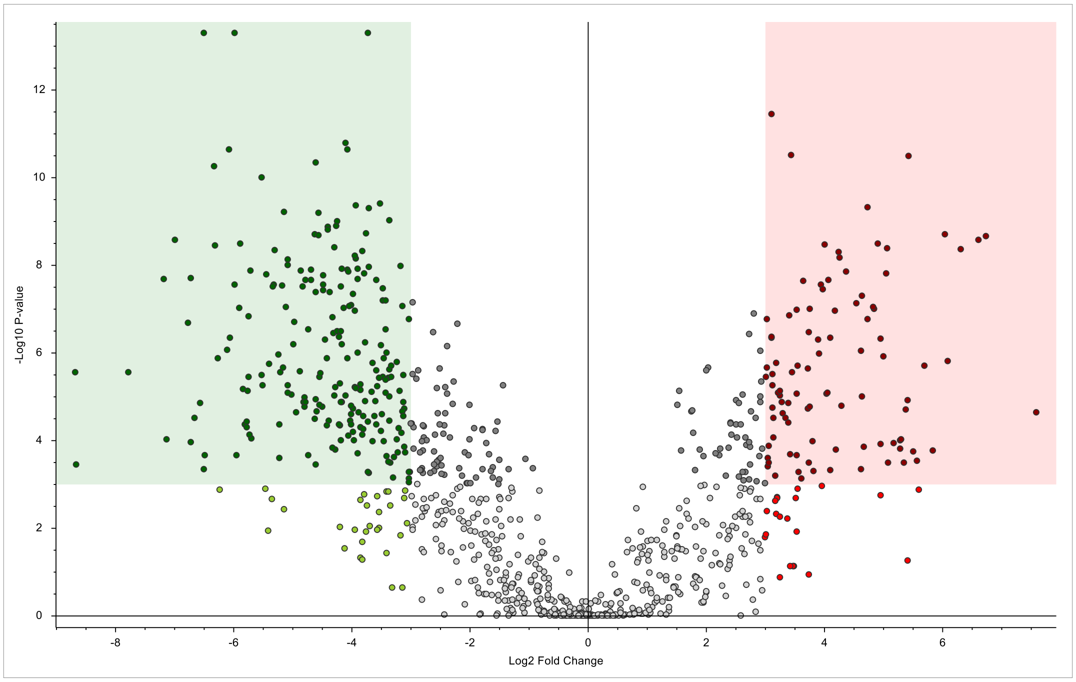

Figure S2. Volcano plot demonstrating the levels of all the metabolites in the Camp_130 vs. Camp_83 ($p<0.001$ and log2 FC>3) in ESI+ mode. In the colored areas, the metabolites that show a statistically significant difference are depicted.

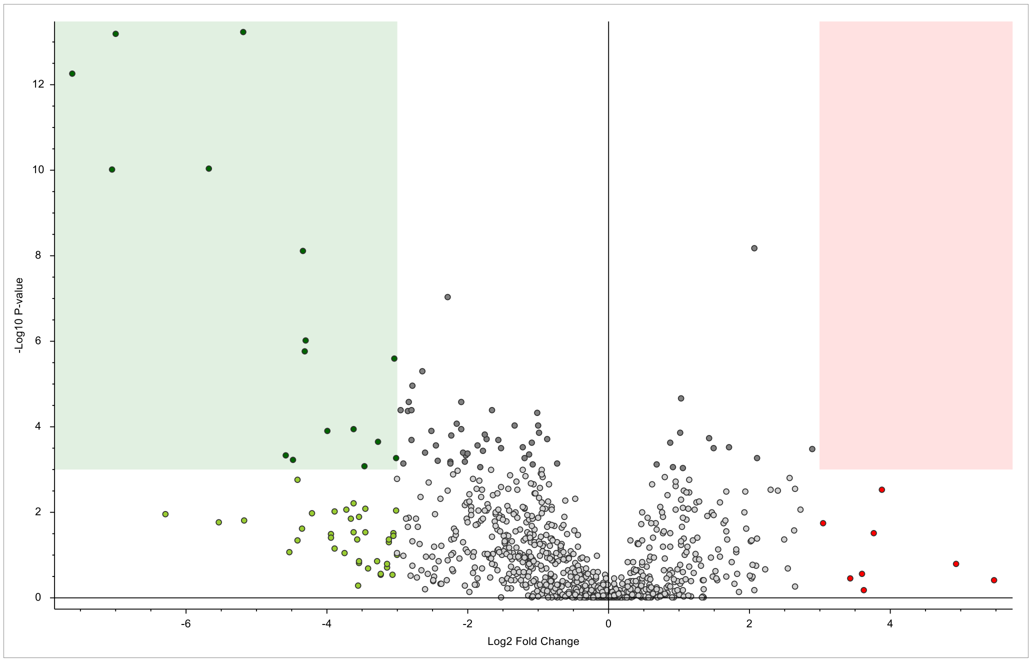

Figure S3. Volcano plot demonstrating the levels of all the metabolites in the Camp_130 vs. Cons1 ($p<0.001$ and log2 FC>3) in ESI+ mode. In the colored areas, the metabolites that show a statistically significant difference are depicted.

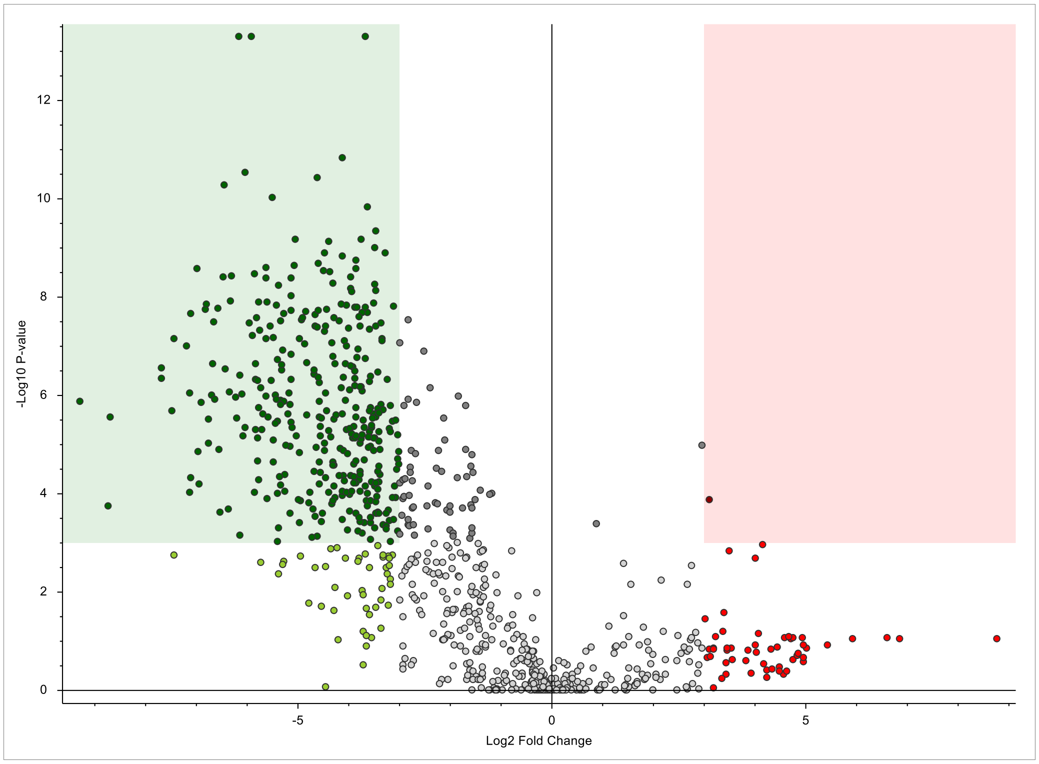

Figure S4. Volcano plot demonstrating the levels of all the metabolites in the Camp_48 vs. Camp83 ($p<0.001$ and log2 FC>3) in ESI+ mode. In the colored areas, the metabolites that show a statistically significant difference are depicted.

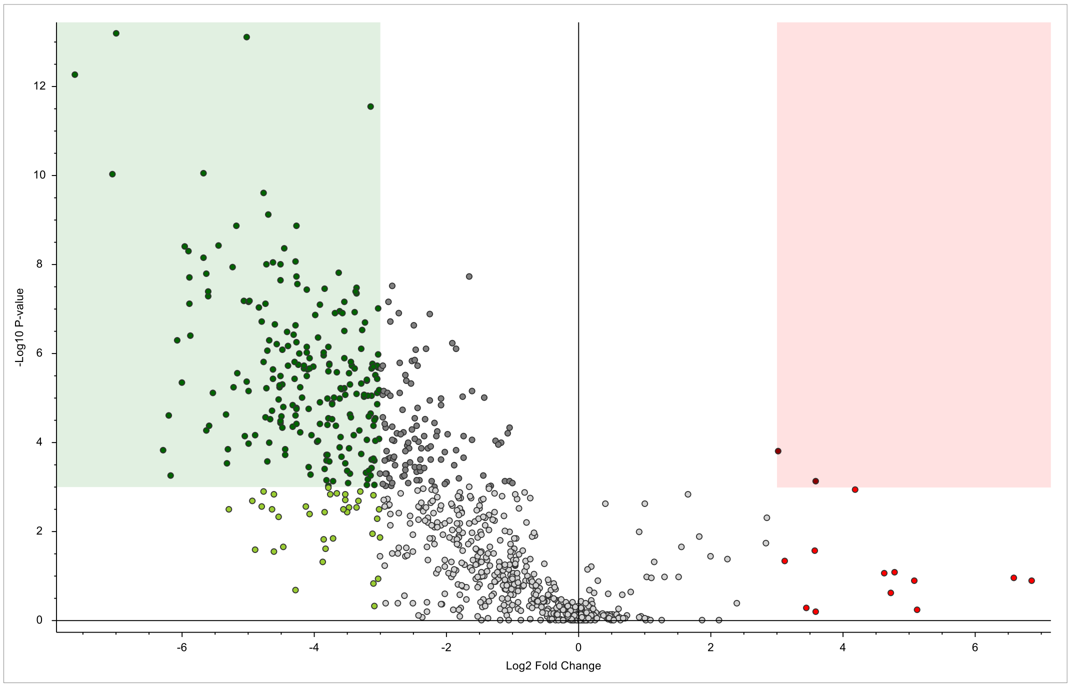

Figure S5. Volcano plot demonstrating the levels of all the metabolites in the Camp_48 vs. Cons1 ($p<0.001$ and log2 FC>3) in ESI+ mode. In the colored areas, the metabolites that show a statistically significant difference are depicted.

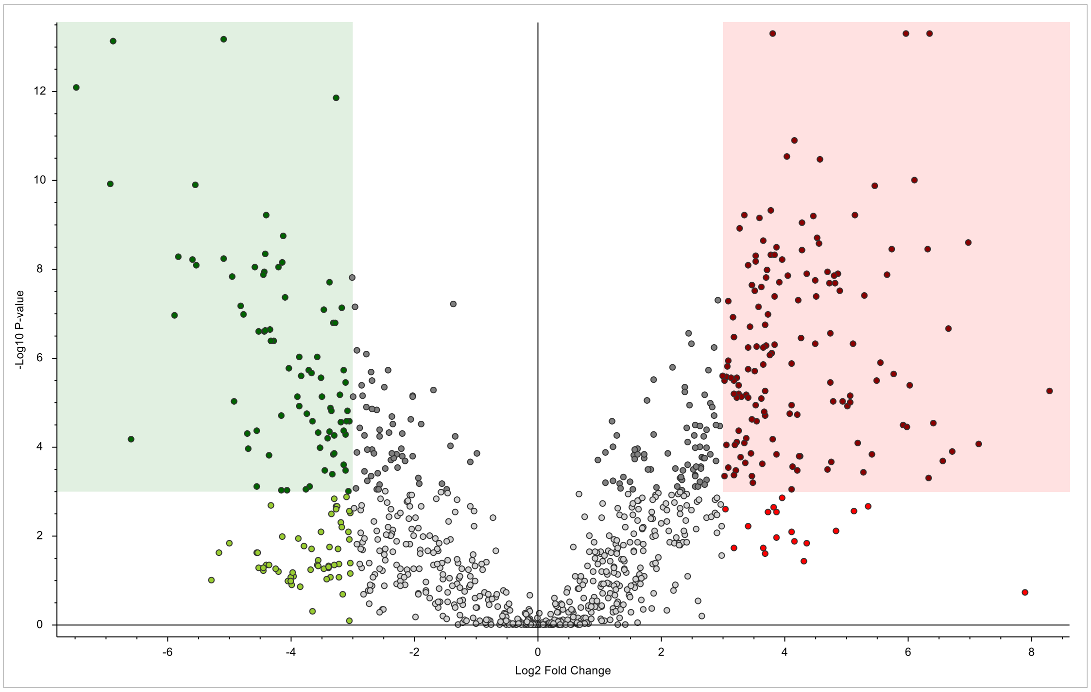

Figure S6. Volcano plot demonstrating the levels of all the metabolites in the Camp_83 vs. Cons1 (p<0.001 and log2 FC>3) in ESI+ mode. In the colored areas, the metabolites that show a statistically significant difference are depicted.

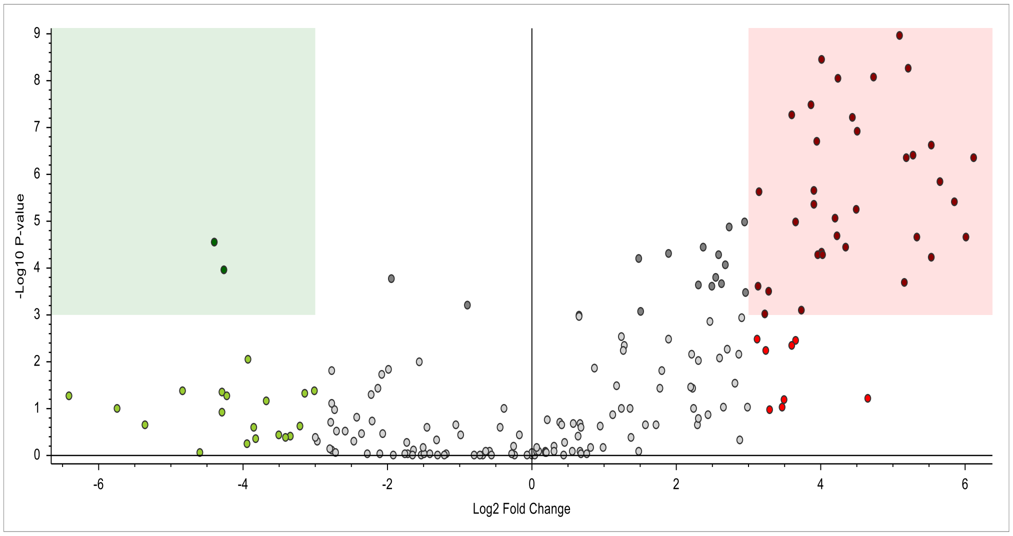

Figure S7. Volcano plot demonstrating the levels of all the metabolites in the Camp_130 vs. Camp_48 (p<0.001 and log2 FC>3) in ESI- mode. In the colored areas, the metabolites that show a statistically significant difference are depicted.

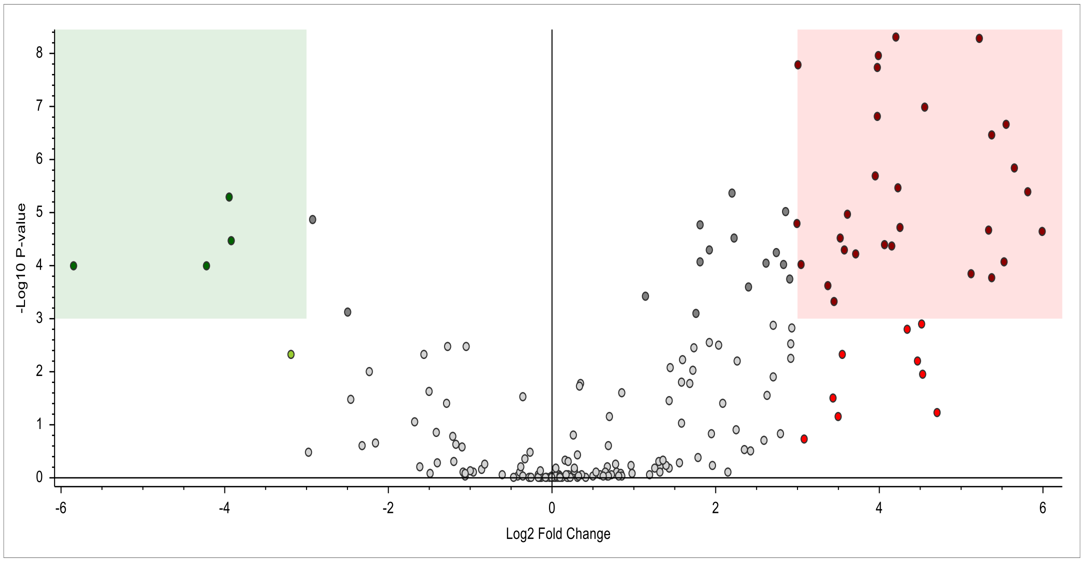

Figure S8. Volcano plot demonstrating the levels of all the metabolites in the Camp_130 vs. Camp_83 (p<0.001 and log2 FC>3) in ESI- mode. In the colored areas, the metabolites that show a statistically significant difference are depicted.

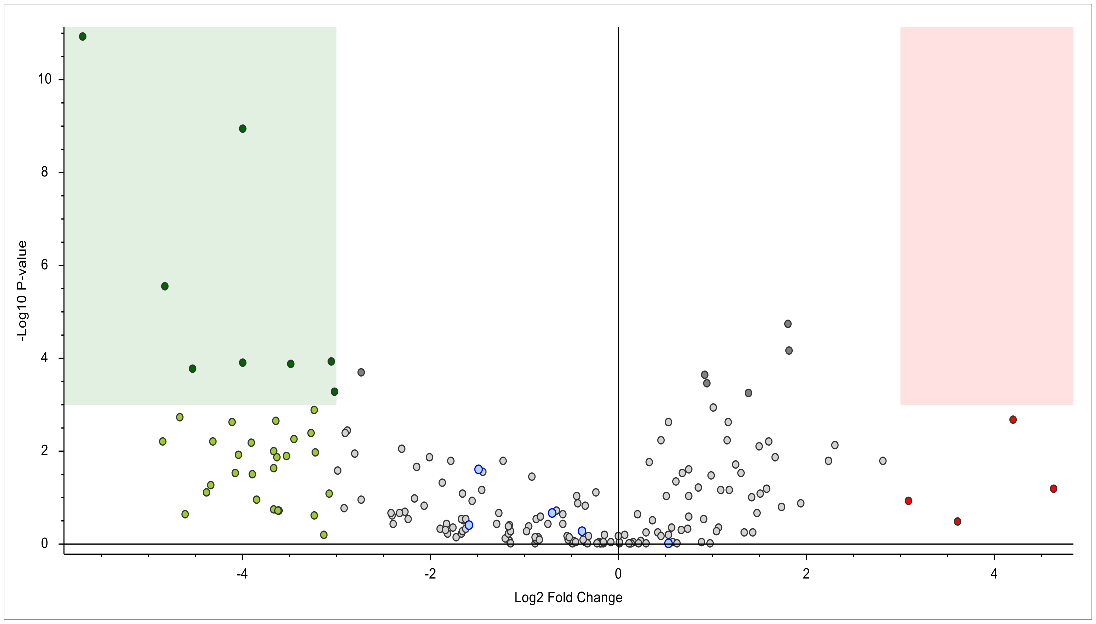

Figure S9. Volcano plot demonstrating the levels of all the metabolites in the Camp_130 vs. Cons1 (p<0.001 and log2 FC>3) in ESI- mode. In the colored areas, the metabolites that show a statistically significant difference are depicted.

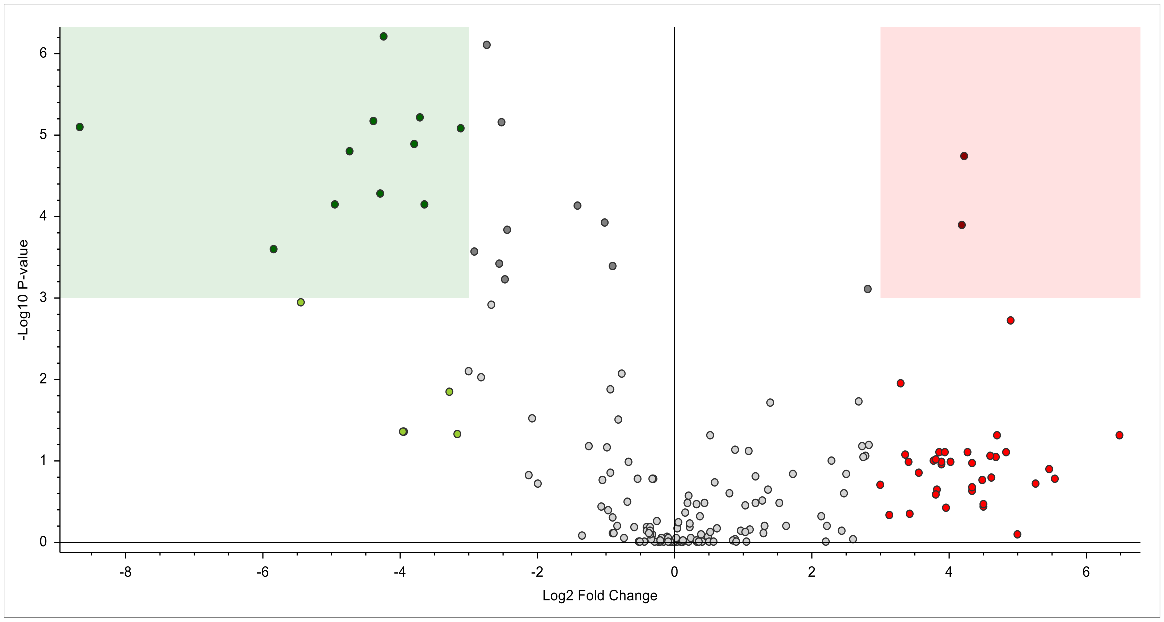

Figure S10. Volcano plot demonstrating the levels of all the metabolites in the Camp_48 vs. Camp83 (p<0.001 and log2 FC>3) in ESI- mode. In the colored areas, the metabolites that show a statistically significant difference are depicted.

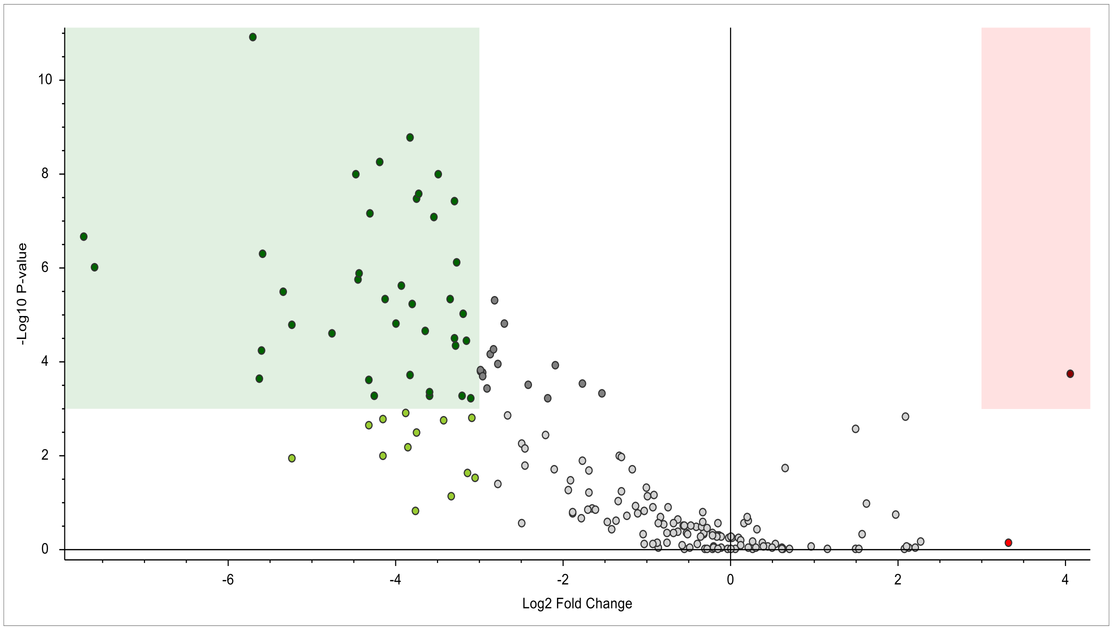

Figure S11. Volcano plot demonstrating the levels of all the metabolites in the Camp_48 vs. Cons1 (p<0.001 and log2 FC>3) in ESI- mode. In the colored areas, the metabolites that show a statistically significant difference are depicted.

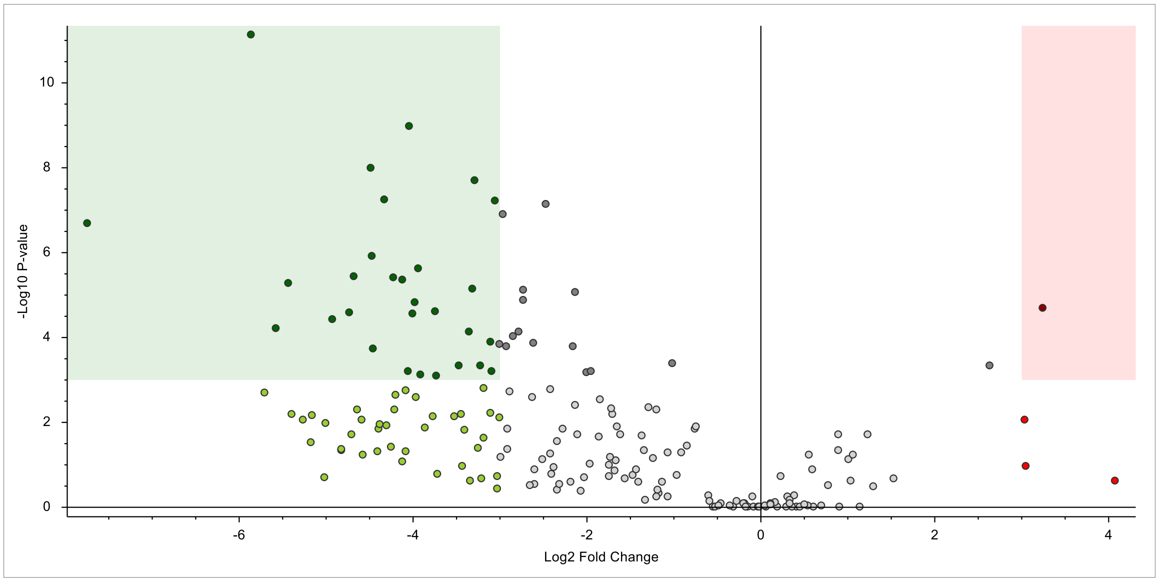

Figure S12. Volcano plot demonstrating the levels of all the metabolites in the Camp_83 vs. Cons1 (p<0.001 and log2 FC>3) in ESI- mode. In the colored areas, the metabolites that show a statistically significant difference are depicted.

Table S1 displays the statistical significance of upregulated identified LC-MS metabolite levels determined by a volcano plot in Camp_130 vs. Camp_48 in positive and negative mode. The names of the identified compounds, their molecular formula and molecular mass as well as the ionization mode in which the substance is detected are presented.

Table S1. LC/MS upregulated features that differed significantly between Camp_130 and Camp_48 in ESI+ and ESI- mode. Data represents directed effected size for each bacteria obtained from volcano plot and significant at P-value ≤ 0.001.

Name	Formula	Molecular Weight	Ionization Mode
4-Nitroaniline	C6 H6 N2 O2	138.04315	Positive
[4-(Aminomethyl)hexahydro-1H-[1,4]oxazino[3,4-c][1,4]oxazin-4-yl]methanol	C9 H18 N2 O3	202.13218	Positive
2-(Trifluoromethyl)-5-pyrimidinylboronic acid	C5 H4 B F3 N2 O2	192.03149	Positive
Trifluorbutane	C4 H7 F3	112.05051	Positive
5-vinyluracil	C6 H6 N2 O2	138.04314	Positive
Methyl 3-amino-3-(3-pyridinyl)propanoate	C9 H12 N2 O2	180.09023	Positive
1-Deoxynojirimycin	C6 H13 N O4	163.08472	Positive
3-(3-Ethoxy-4-hydroxy-1-pyrrolidinyl)propanoic acid	C9 H17 N O4	203.11616	Positive
4,4'-Dicyanobenzhydrol	C15 H10 N2 O	234.07838	Positive
1,1',1''-Phosphinetriyltris(1H-imidazole)	C9 H9 N6 P	232.06285	Positive

Methyl 3-acetamido-6-[(3,6-diacetamidohexanoyl)amino]hexanoate	C19 H34 N4 O6	414.24828	Positive
O-Ethyl S-methyl methylphosphonothioate	C4 H11 O2 P S	154.02119	Positive
5,6-Diamino-2-thiouracil	C4 H6 N4 O S	158.02586	Positive
(3-Amino-1-azetidinyl)(4-methyl-1,2,3-thiadiazol-5-yl)methanone	C7 H10 N4 O S	198.05725	Positive
Allantoic acid	C4 H8 N4 O4	176.05385	Positive
(4-Cyclohexyl-1-piperazinyl)(4-nitrophenyl)methanone	C17 H23 N3 O3	317.17459	Positive
Methyl N-decanoylglycylprolyltryptophylleucinate	C35 H53 N5 O6	639.39663	Positive
[(4-Amino-5-propyl-4H-1,2,4-triazol-3-yl)sulfanyl]acetic acid	C7 H12 N4 O2 S	216.06781	Positive
N-(3-Methyl-2-buten-1-yl)-2-(methylsulfanyl)-7H-purin-6-amine	C11 H15 N5 S	249.10516	Positive
2-Amino-5-{[(4,5-dihydroxy-2-cyclopenten-1-yl)amino]methyl}-4H-pyrrolo[2,3-d]pyrimidin-4-on	C12 H13 N5 O3	275.10108	Positive
1-[1-(Methylsulfonyl)-3-pyrrolidinyl]-1H-1,2,3-triazole-4-carbaldehyde	C8 H12 N4 O3 S	244.06276	Positive
2-[(1,2-Dimethyl-4-nitro-1H-imidazol-5-yl)amino]ethanol	C7 H12 N4 O3	200.0903	Positive
Pentoxyl	C6 H8 N2 O3	156.05379	Positive
Methyl N-(methoxyacetyl)-2-methylalaninate	C8 H15 N O4	189.10054	Positive
6-(4-(Methylsulfonyl)piperazin-1-yl)pyridin-3-amine	C10 H16 N4 O2 S	256.09917	Positive
2-(2-AMINOETHYLAMINO)-5-NITROPYRIDINE	C7 H10 N4 O2	182.07987	Positive
3-(3-Ethoxy-4-nitro-1H-pyrazol-1-yl)-1-propanamine	C8 H14 N4 O3	214.10598	Positive
L-alpha-Aspartyl-L-phenylalanine	C13 H16 N2 O5	280.10647	Positive
5-Amino-3-(methylsulfanyl)-1H-pyrazole-4-carboxamide	C5 H8 N4 O S	172.04159	Positive
2-Nitrophenyl 5-{1-[4-(2-methyl-2-propanyl)benzoyl]-2-oxohexahydro-1H-thieno[3,4-d]imidazol-4-yl}pentanoate	C27 H31 N3 O6 S	525.19297	Positive
2-Methyl-2-propanyl N-[(4-{[5-({[(2-methyl-2-propanyl)oxy]carbonyl}amino)pentyl]carbamoyl}-1H-imidazol-5-yl)carbonyl]glycinate	C21 H35 N5 O6	453.25983	Positive
LY2090314	C28 H25 F N6 O3	512.19846	Positive
Methyl (2S,2aS,3a'R,4a'R,5aR,12bR,12b'R,12c'R,15aR,15a'S,17a'S)-12c'-hydroxy-10'-methoxy-2',3',4,4a',5,6,8,12c',13,13',14,14',17',17a'-tetradecahydro-4'H,5'H,16'H-spiro[furo[2',3':7,8]indolizino[8,1-c d]carbazole-2,6'-furo[2',3':7,8]indolizino[8,1-cd]pyrido[1,2,3-lm]carbazole]-7-carboxylate	C43 H50 N4 O6	718.37003	Positive
Acetyl Proline	C7 H11 N O3	157.0742	Positive
1-Azido-2-{2-[2-(2-isothiocyanatoethoxy)ethoxy]ethoxy}ethane	C9 H16 N4 O3 S	260.09403	Positive
2-{2-[1-Amino-2-(1H-1,2,4-triazol-1-yl)ethylidene]hydrazino}-2-oxoacetamide	C6 H9 N7 O2	211.08245	Positive
2-(Hydroxymethyl)-2-(1H-tetrazol-1-yl)-1,3-propanediol	C5 H10 N4 O3	174.0746	Positive

Iridodial glucoside tetraacetate	C24 H34 O11	498.21039	Positive
4-[4-(3-Methoxypropyl)-5-methyl-4H-1,2,4-triazol-3-yl]-1-methyl-1H-pyrazol-5-amine	C11 H18 N6 O	250.15479	Positive
Enadenine	C10 H13 N5	203.1175	Positive
L-Leucyl-L-alanyl-L-proline	C14 H25 N3 O4	299.18503	Positive
(4R,6R)-t-Butyl-6-(2-aminoethyl)-2,2-dimethyl-1,3-dioxane-4-acetate	C14 H27 N O4	273.1945	Positive
1-[4-(1H-Purin-6-yl)-2-morpholinyl]methanamine	C10 H14 N6 O	234.12351	Positive
3-Phenylindeno[1,2-c]pyrazol-4(1H)-one	C16 H10 N2 O	246.07827	Positive
ala-leu-ala-pro	C17 H30 N4 O5	370.22215	Positive
(3beta,16alpha,22alpha)-16,28-Dihydroxy-22-{[(2Z)-2-methyl-2-butenoyl]oxy}olean-12-en-3-yl beta-D-galactopyranosyl-(1->2)-[beta-D-xylopyranosyl-(1->2)-alpha-L-arabinopyranosyl-(1->3)]-beta-D-glucopyranosiduronic acid	C57 H90 O24	1158.58213	Positive
3-(4-Pyridinyl)-5H-indeno[1,2-c]pyridazin-5-one	C16 H9 N3 O	259.0739	Positive
3-hydroxydodecanoylcarnitine	C19 H37 N O5	359.26677	Negative
1-Isopropoxy-2,8,9-trioxa-5-aza-1-silabicyclo[3.3.3]undecane	C9 H19 N O4 Si	233.10798	Negative
N-(PHENYLMETHYLIDENE)BENZENESULFONAMIDE	C13 H11 N O2 S	245.05056	Negative
2-Acetamido-3-O-[(1R)-1-carboxyethyl]-2-deoxy-1-O-phosphono-alpha-D-glucopyranose	C11 H20 N O11 P	373.0769	Negative
Myriocin	C21 H39 N O6	401.27722	Negative
4-NITROPHENYL PHENYL SULFIDE	C12 H9 N O2 S	231.0348	Negative
Ethyl 2-(benzoylamino)-7-oxo-4,5,6,7-tetrahydro-1-benzothiophene-3-carboxylate	C18 H17 N O4 S	343.08754	Negative
N,N’-Di-Boc-S-methylisothiourea	C12 H22 N2 O4 S	290.13007	Negative
val-leu-gly-lys	C19 H37 N5 O5	415.27912	Negative
5-Oxidanidyl-5-oxidanylidenenorvalylarginine	C11 H20 N5 O5	302.14751	Negative
4-Methyl-2-{(E)-[4-(methylsulfanyl)benzylidene]amino}phenol	C15 H15 N O S	257.08702	Negative
9-Oxo-9,10-dihydro-2-acridinesulfonic acid	C13 H9 N O4 S	275.02508	Negative
Phosphinic acid, diphenyl-, anhydride	C24 H20 O3 P2	418.08711	Negative
Dimethyl(2-methyl-2-propanyl)silyl N-[dimethyl(2-methyl-2-propanyl)silyl]isoleucinate	C18 H41 N O2 Si2	359.2667	Negative

Table S2 displays the statistical significance of downregulated identified LC-MS metabolite levels determined by a volcano plot in Camp_130 vs. Camp_48 in positive and negative mode. The names of the identified compounds, their molecular formula and molecular mass as well as the ionization mode in which the substance is detected are presented.

Table S2. LC/MS downregulated features that differed significantly between Camp_130 and Camp_48 in ESI+ and ESI- mode. Data represents directed effected size for each bacteria obtained from volcano plot and significant at P-value ≤ 0.001.

Name	Formula	Molecular Weight	Ionization Mode
6-Azido-6-deoxy-1,2-O-isopropylidene-alpha-D-glucofuranose	C9 H15 N3 O5	245.10148	Positive
Methyl-4,6-dideoxy-4-[(2,4-dihydroxybutanoyl)amino]hexopyranoside	C11 H21 N O7	279.13145	Negative
N-[5,5-Bis(ethylsulfanyl)-3,4-dihydroxy-2-pentanyl]acetamide	C11 H23 N O3 S2	281.1107	Negative

Table S3 displays the statistical significance of upregulated identified LC-MS metabolite levels determined by a volcano plot in Camp_130 vs. Camp_83 in positive and negative mode. The names of the identified compounds, their molecular formula and molecular mass as well as the ionization mode in which the substance is detected are presented.

Table S3. LC/MS upregulated features that differed significantly between Camp_130 and Camp_83 in ESI+ and ESI- mode. Data represents directed effected size for each bacteria obtained from volcano plot and significant at P-value ≤ 0.001.

Name	Formula	Molecular Weight	Ionization Mode
4-Nitroaniline	C6 H6 N2 O2	138.04315	Postive
N-Acetyl-L-leucine	C8 H15 N O3	173.1056	Postive
5-vinyluracil	C6 H6 N2 O2	138.04314	Postive
N-Ethyl-N-(4-nitrobenzyl)-1,2-ethanediamine	C11 H17 N3 O2	223.13247	Postive
1-Deoxynojirimycin	C6 H13 N O4	163.08472	Postive
5,6-Diamino-2-thiouracil	C4 H6 N4 O S	158.02586	Postive
1-(7-Hydroxy-6-methoxy-3,4-dihydro-2(1H)-isoquinolinyl)-1-propanone	C13 H17 N O3	235.12127	Postive
(3-Amino-1-azetidinyl)(4-methyl-1,2,3-thiadiazol-5-yl)methanone	C7 H10 N4 O S	198.05725	Postive
Quinuclidinol	C7 H13 N O	127.10009	Postive
N-(3-Methyl-2-buten-1-yl)-2-(methylsulfanyl)-7H-purin-6-amine	C11 H15 N5 S	249.10516	Postive
pentoxyl	C6 H8 N2 O3	156.05379	Postive
Octahydro-1,6,7,8-indolizinetetrol	C8 H15 N O4	189.10034	Postive

2-(2-AMINOETHYLAMINO)-5-NITROPYRIDINE	C7 H10 N4 O2	182.07987	Postive
3-(3-Ethoxy-4-nitro-1H-pyrazol-1-yl)-1-propanamine	C8 H14 N4 O3	214.10598	Postive
2-Amino-4-(methylamino)butanoic acid	C5 H12 N2 O2	132.09017	Postive
5-Amino-3-(methylsulfanyl)-1H-pyrazole-4-carboxamide	C5 H8 N4 O S	172.04159	Postive
1-Azido-2-{2-[2-(2-isothiocyanatoethoxy)ethoxy]ethoxy}ethane	C9 H16 N4 O3 S	260.09403	Postive
2-(Hydroxymethyl)-2-(1H-tetrazol-1-yl)-1,3-propanediol	C5 H10 N4 O3	174.0746	Postive
Iridodial glucoside tetraacetate	C24 H34 O11	498.21039	Postive
N-(7-Amino-6-methyl-7H-[1,2,4]triazolo[4,3-b][1,2,4]triazol-3-yl)acetamide	C6 H9 N7 O	195.08761	Postive
L-Leucyl-L-alanyl-L-proline	C14 H25 N3 O4	299.18503	Postive
(4R,6R)-t-Butyl-6-(2-aminoethyl)-2,2-dimethyl-1,3-dioxane-4-acetate	C14 H27 N O4	273.1945	Postive
1-[4-(1H-Purin-6-yl)-2-morpholinyl]methanamine	C10 H14 N6 O	234.12351	Postive
3-Phenylindeno[1,2-c]pyrazol-4(1H)-one	C16 H10 N2 O	246.07827	Postive
ala-leu-ala-pro	C17 H30 N4 O5	370.22215	Postive
Iridodial glucoside tetraacetate	C24 H34 O11	498.20809	Postive
3-hydroxydodecanoylcarnitine	C19 H37 N O5	359.26677	Negative
1-Isopropoxy-2,8,9-trioxa-5-aza-1-silabicyclo[3.3.3]undecane	C9 H19 N O4 Si	233.10798	Negative
2-Acetamido-3-O-[(1R)-1-carboxyethyl]-2-deoxy-1-O-phosphono-alpha-D-glucopyranose	C11 H20 N O11 P	373.0769	Negative
Myriocin	C21 H39 N O6	401.27722	Negative
Ethyl 2-(benzoylamino)-7-oxo-4,5,6,7-tetrahydro-1-benzothiophene-3-carboxylate	C18 H17 N O4 S	343.08754	Negative
Methyl N-butyrylvalinate	C10 H19 N O3	201.13569	Negative
val-leu-gly-lys	C19 H37 N5 O5	415.27912	Negative
5-Oxidanidyl-5-oxidanylidenenorvalylarginine	C11 H20 N5 O5	302.14751	Negative
Phosphinic acid, diphenyl-, anhydride	C24 H20 O3 P2	418.08711	Negative

Dimethyl(2-methyl-2-propanyl)silyl N-[dimethyl(2-methyl-2-propanyl)silyl]isoleucinate	C18 H41 N O2 Si2	359.2667	Negative
4-mercaptophenylboronic acid	C6 H7 B O2 S	154.02548	Negative

Table S4 displays the statistical significance of downregulated identified LC-MS metabolite levels determined by a volcano plot in Camp_130 vs. Camp_83 in positive and negative mode. The names of the identified compounds, their molecular formula and molecular mass as well as the ionization mode in which the substance is detected are presented.

Table S4. LC/MS downregulated features that differed significantly between Camp_130 and Camp_83 in ESI+ and ESI- mode. Data represents directed effected size for each bacteria obtained from volcano plot and significant at P-value ≤ 0.001.

Name	Formula	Molecular Weight	Ionization Mode
Prolylleucine (Pro-Leu)	C11 H20 N2 O3	228.14763	Positive
REPOSAL	C14 H18 N2 O3	262.132	Positive
Pro-Ileu	C11 H20 N2 O3	228.14774	Positive
2-[(6,6-Dimethyl-2-morpholinyl)methoxy]-N,N-dimethylacetamide	C11 H22 N2 O3	230.16337	Positive
Leu-Leu	C12 H24 N2 O3	244.17901	Positive
Val-pro	C10 H18 N2 O3	214.13205	Positive
Val-Leu	C11 H22 N2 O3	230.16335	Positive
D-lysopine	C9 H18 N2 O4	218.12696	Positive
tert-Butyl N-[1-(aminocarbonyl)-3-methylbutyl]carbamate	C11 H22 N2 O3	230.16344	Positive
2-(2-amino-3-methylbutanamido)-3-phenylpropanoic acid	C14 H20 N2 O3	264.14782	Positive
L-gamma-Glutamyl-L-leucine	C11 H20 N2 O5	260.13762	Positive
N2-Acetyl-L-lysine	C8 H16 N2 O3	188.11638	Positive
D-lysopine	C9 H18 N2 O4	218.12699	Positive
Leu-Tyr	C15 H22 N2 O4	294.15839	Positive

3-{[(4-Methoxy-1-piperidinyl)carbonyl]amino}benzoic acid	C14 H18 N2 O4	278.12713	Positive
Tert-Butyl 4-(3-Amino-2-Hydroxypropyl)Piperazine-1-Carboxylate	C12 H25 N3 O3	259.19	Positive
2-Nitroso-6,7-dimethoxytetrahydroisoquinoline	C11 H14 N2 O3	222.10073	Positive
N-(3-Nitrophenyl)octanamide	C14 H20 N2 O3	264.14786	Positive
2-Amino-4-methylpyrimidine	C5 H7 N3	109.06351	Positive
Leu-pro	C11 H20 N2 O3	228.14779	Positive
met-val	C10 H20 N2 O3 S	248.11979	Positive
tert-Butyl hydrazodiformate	C10 H20 N2 O4	232.14262	Positive
N-Boc-serinol	C8 H17 N O4	191.11599	Positive
val-arg	C11 H23 N5 O3	273.18047	Positive
[4-(Aminomethyl)hexahydro-1H-[1,4]oxazino[3,4-c][1,4]oxazin-4-yl]methanol	C9 H18 N2 O3	202.13218	Positive
Val-pro	C10 H18 N2 O3	214.13215	Positive
Val-Gln	C10 H19 N3 O4	245.13791	Positive
2,2'-[(6-Aminohexyl)imino]diacetic acid	C10 H20 N2 O4	232.14266	Positive
9-mercaptodethiobiotin	C10 H18 N2 O3 S	246.10421	Positive
[4-(Aminomethyl)hexahydro-1H-[1,4]oxazino[3,4-c][1,4]oxazin-4-yl]methanol	C9 H18 N2 O3	202.13215	Positive
Ala-Tyr	C12 H16 N2 O4	252.1113	Positive
5-Methoxy-1-(1-methyl-4-piperidinyl)-4-oxo-1,4-dihydro-2-pyridinecarboxylic acid	C13 H18 N2 O4	266.12706	Positive
Leu-arg	C12 H25 N5 O3	287.19616	Positive
beta-Ala-Phe	C12 H16 N2 O3	236.11649	Positive
Cadralazine	C12 H21 N5 O3	283.165	Positive
2-(4-Morpholinylmethyl)-3-(2-oxopropyl)-4(3H)-quinazolinone	C16 H19 N3 O3	301.14321	Positive
Ala-Tyr	C12 H16 N2 O4	252.11129	Positive
Lys-phe	C15 H23 N3 O3	293.17443	Positive

5-Methoxy-1-(1-methyl-4-piperidinyl)-4-oxo-1,4-dihydro-2-pyridinecarboxylic acid	C13 H18 N2 O4	266.12723	Positive
Val-Met	C10 H20 N2 O3 S	248.11984	Positive
N,N'-Di-Boc-guanidine	C11 H21 N3 O4	259.15371	Positive
Leu-arg	C12 H25 N5 O3	287.19623	Positive
Ethyl 1-(4-amino-6-oxo-1,6-dihydro-2-pyrimidinyl)-4-piperidinecarboxylate	C12 H18 N4 O3	266.13848	Positive
ala-met	C8 H16 N2 O3 S	220.08859	Positive
(2S)-3-Methyl-2-{[(2S,3R,4R,5R)-2,3,4,5,6-pentahydroxyhexyl]amino}butanoic acid	C11 H23 N O7	281.1462	Positive
Lys-Lys	C12 H26 N4 O3	274.20097	Positive
tert-butyl 3-[(N'-hydroxycarbamimidoyl)methyl]piperidine-1-carboxylate	C12 H23 N3 O3	257.1746	Positive
ile-pro-lys	C17 H32 N4 O4	356.24305	Positive
His-Leu	C12 H20 N4 O3	268.15398	Positive
Di-Tert-Butyl 2-Oxo-1,8,11-Triazaspiro[5.6]Dodecane-8,11-Dicarboxylate	C19 H33 N3 O5	383.24277	Positive
asp-arg	C10 H19 N5 O5	289.13835	Positive
Leu-pro	C11 H20 N2 O3	228.14744	Positive
gln-leu-leu-pro	C22 H39 N5 O6	469.29089	Positive
7-(4-Methyl-1-piperazinyl)[1,3]thiazolo[4,5-d]pyrimidin-5-amine	C10 H14 N6 S	250.09901	Positive
LYSINOALANINE, (S,R)-	C9 H19 N3 O4	233.13792	Positive
Ethyl 4-[4-({2-[(3-ethoxy-3-oxo-1-phenylpropyl)amino]-2-oxoethyl}amino)-4-oxobutanoyl]-1-piperazinecarboxylate	C24 H34 N4 O7	490.24433	Positive
2-Amino-4-(methylsulfonyl)-N-(tetrahydro-2H-pyran-4-yl)butanamide	C10 H20 N2 O4 S	264.11467	Positive
Leu-Tyr	C15 H22 N2 O4	294.15852	Positive
Tris(trimethylsilyl)amine	C9 H27 N Si3	233.14598	Positive
Ethyl {[4-(dimethylamino)-6-(methoxyamino)-1,3,5-triazin-2-yl]oxy}acetate	C10 H17 N5 O4	271.12836	Positive
Lys-Pro	C11 H21 N3 O3	243.15904	Positive

1-BOC-ISONIPECOTIC ACID HYDRAZIDE	C11 H21 N3 O3	243.15874	Positive
2-(4-Isopropyl-4-methyl-5-oxo-2-imidazolin-2-yl)nicotinic acid	C13 H15 N3 O3	261.11181	Positive
1H-Imidazole-4,5-dicarbohydrazide	C5 H8 N6 O2	184.07155	Positive
Dibutyl(phenyl)phosphine sulfide	C14 H23 P S	254.12468	Positive
thr-arg	C10 H21 N5 O4	275.15974	Positive
N,N',N''-Tri-Boc-guanidine	C16 H29 N3 O6	359.2063	Positive
4-Methoxy-N-{[3-(2-pyrazinyl)-1H-1,2,4-triazol-5-yl]methyl}aniline	C14 H14 N6 O	282.12207	Positive
N-Hydroxy-2-[4-(N-hydroxycarbamimidoyl)phenyl]-1H-benzimidazole-5-carboximidamide	C15 H14 N6 O2	310.11687	Positive
ile-val-lys	C17 H34 N4 O4	358.25864	Positive
North-Methanocarbathymidine (**N-MCT**)	C12 H16 N2 O4	252.11131	Positive
3-Cyanopropyltriethoxysilane	C10 H21 N O3 Si	231.12988	Positive
Methyl [3-(3,4-diethoxyphenyl)-1H-1,2,4-triazol-1-yl]acetate	C15 H19 N3 O4	305.13811	Positive
val-ile-arg	C17 H34 N6 O4	386.26518	Positive
Biotinamidocaproyl hydrazide	C16 H29 N5 O3 S	371.19945	Positive
9-mercaptodethiobiotin	C10 H18 N2 O3 S	246.10429	Positive
Trialanine	C9 H17 N3 O4	231.12213	Positive
2-(benzoylamino)-3-phenylpropanoic acid	C16 H15 N O3	269.1039	Positive
asn-trp-arg	C21 H30 N8 O5	474.23395	Positive
N,N'-Di-Boc-guanidine	C11 H21 N3 O4	259.15383	Positive
Roblitinib	C25 H30 N8 O4	506.23833	Positive
(S)-4-(4-(5-(Aminomethyl)-2-oxooxazolidin-3-yl)phenyl)morpholin-3-one	C14 H17 N3 O4	291.12258	Positive
6-{3-[(progesterone-4-yl)thiopropionyl]amino}hexanoic acid	C30 H45 N O5 S	531.30265	Positive
1,3,3-Trimethyl-6'-morpholinospiro[indoline-2,3'-naphtho[2,1-b][1,4]oxazine]	C26 H27 N3 O2	413.20922	Positive

tris(trimethylsilyl)phosphane	C9 H27 P Si3	250.11603	Positive
Dimepiperate	C7 H17 N7 O4	263.13466	Negative
6-[2-(Acetyloxymethyl)-4,5,6-trihydroxyoxan-3-yl]oxy-3,4,5-trihydroxyoxane-2-carboxylic acid	C14 H22 O13	398.10615	Negative

Table S5 displays the statistical significance of downregulated identified LC-MS metabolite levels determined by a volcano plot in Camp_130 vs. Cons1 in positive and negative mode. The names of the identified compounds, their molecular formula and molecular mass as well as the ionization mode in which the substance is detected are presented.

Table S5. LC/MS downregulated features that differed significantly between Camp_130 and Cons1 in ESI+ and ESI- mode. Data represents directed effected size for each bacteria obtained from volcano plot and significant at P-value ≤ 0.001.

Name	Formula	Molecular Weight	Ionization Mode
2,2'-[(6-Aminohexyl)imino]diacetic acid	C10 H20 N2 O4	232.14266	Positive
Leu-arg	C12 H25 N5 O3	287.19616	Positive
Ileu-arg	C12 H25 N5 O3	287.19623	Positive
N-Ethyl-N'-isopropyl-6-(4-morpholinyl)-1,3,5-triazine-2,4-diamine	C12 H22 N6 O	266.1862	Positive
N-(2-Azidoethyl)-N-{2-[{2-[methyl(propionyl)amino]ethyl}(propionyl)amino]ethyl}propanamide	C16 H30 N6 O3	354.23857	Positive
4-Methyl-1H-[1,2,3]triazolo[4,5-d]pyridazin-7-ol	C5 H5 N5 O	151.04971	Positive
Glaurin	C16 H32 O4	288.22986	Negative
6-[2-(Acetyloxymethyl)-4,5,6-trihydroxyoxan-3-yl]oxy-3,4,5-trihydroxyoxane-2-carboxylic acid	C14 H22 O13	398.10615	Negative

Table S6 displays the statistical significance of upregulated identified LC-MS metabolite levels determined by a volcano plot in Camp_48 vs. Camp_83 in positive and negative mode. The names of the identified compounds, their molecular formula and molecular mass as well as the ionization mode in which the substance is detected are presented.

Table S6. LC/MS upregulated features that differed significantly between Camp_48 and Camp_83 in ESI+ and ESI- mode. Data represents directed effected size for each bacteria obtained from volcano plot and significant at P-value ≤ 0.001.

Name	Formula	Molecular Weight	Ionization Mode
3-(3,4-Dimethoxyphenyl)-1-ethyl-6-methylpyrimido[5,4-e][1,2,4]triazine-5,7(1H,6H)-dione	C16 H17 N5 O4	343.12712	Positive
Methyl 4,6-dideoxy-4-[(2,4-dihydroxybutanoyl)amino]hexopyranoside	C11 H21 N O7	279.13145	Negative
N-[5,5-Bis(ethylsulfanyl)-3,4-dihydroxy-2-pentanyl]acetamide	C11 H23 N O3 S2	281.1107	Negative

Table S7 displays the statistical significance of downregulated identified LC-MS metabolite levels determined by a volcano plot in Camp_48 vs. Camp_83 in positive and negative mode. The names of the identified compounds, their molecular formula and molecular mass as well as the ionization mode in which the substance is detected are presented.

Table S7. LC/MS downregulated features that differed significantly between Camp_48 and Camp_83 in ESI+ and ESI- mode. Data represents directed effected size for each bacteria obtained from volcano plot and significant at P-value ≤ 0.001.

Name	Formula	Molecular Weight	Ionization Mode
Prolylleucine (Pro-Leu)	C11 H20 N2 O3	228.14763	Positive
REPOSAL	C14 H18 N2 O3	262.132	Positive
tert-Butyl N-[1-(aminocarbonyl)-3-methylbutyl]carbamate	C11 H22 N2 O3	230.1633	Positive
Leu-Ileu	C11 H20 N2 O3	228.14774	Positive
2-[(6,6-Dimethyl-2-morpholinyl)methoxy]-N,N-dimethylacetamide	C11 H22 N2 O3	230.16337	Positive
Leu-Leu	C12 H24 N2 O3	244.17901	Positive
Val-pro	C10 H18 N2 O3	214.13205	Positive
Val-Leu	C11 H22 N2 O3	230.16335	Positive
D-lysopine	C9 H18 N2 O4	218.12696	Positive
Leu-Leu	C12 H24 N2 O3	244.17908	Positive
tert-Butyl N-[1-(aminocarbonyl)-3-methylbutyl]carbamate	C11 H22 N2 O3	230.16344	Positive

2-(2-amino-3-methylbutanamido)-3-phenylpropanoic acid	C14 H20 N2 O3	264.14782	Positive
N-[(2-Methyl-2-propanyl)carbamoyl]valine	C10 H20 N2 O3	216.14777	Positive
L-gamma-Glutamyl-L-leucine	C11 H20 N2 O5	260.13762	Positive
N2-Acetyl-L-lysine	C8 H16 N2 O3	188.11638	Positive
Leu-Tyr	C15 H22 N2 O4	294.15839	Positive
3-{[(4-Methoxy-1-piperidinyl)carbonyl]amino}benzoic acid	C14 H18 N2 O4	278.12713	Positive
Tert-Butyl 4-(3-Amino-2-Hydroxypropyl)Piperazine-1-Carboxylate	C12 H25 N3 O3	259.19	Positive
2-Nitroso-6,7-dimethoxytetrahydroisoquinoline	C11 H14 N2 O3	222.10073	Positive
N-(3-Nitrophenyl)octanamide	C14 H20 N2 O3	264.14786	Positive
2-Amino-4-methylpyrimidine (UV6485000)	C5 H7 N3	109.06351	Positive
Leu-pro	C11 H20 N2 O3	228.14779	Positive
met-val	C10 H20 N2 O3 S	248.11979	Positive
tert-Butyl hydrazodiformate	C10 H20 N2 O4	232.14262	Positive
N-Boc-serinol	C8 H17 N O4	191.11599	Positive
val-arg	C11 H23 N5 O3	273.18047	Positive
[4-(Aminomethyl)hexahydro-1H-[1,4]oxazino[3,4-c][1,4]oxazin-4-yl]methanol	C9 H18 N2 O3	202.13218	Positive
Val-pro	C10 H18 N2 O3	214.13215	Positive
DL-Ala-DL-Leu	C9 H18 N2 O3	202.13218	Positive
Ro 20-1724	C15 H22 N2 O3	278.16344	Positive
Val-Gln	C10 H19 N3 O4	245.13791	Positive
2-(Trifluoromethyl)-5-pyrimidinylboronic acid	C5 H4 B F3 N2 O2	192.03149	Positive
2,2'-[(6-Aminohexyl)imino]diacetic acid	C10 H20 N2 O4	232.14266	Positive
3,5-Dimethoxy-N-[2-(4-morpholinyl)ethyl]benzamide	C15 H22 N2 O4	294.15855	Positive
9-mercaptodethiobiotin	C10 H18 N2 O3 S	246.10421	Positive

met-ile	C11 H22 N2 O3 S	262.1356	Positive
[4-(Aminomethyl)hexahydro-1H-[1,4]oxazino[3,4-c][1,4]oxazin-4-yl]methanol	C9 H18 N2 O3	202.13215	Positive
Ala-Tyr	C12 H16 N2 O4	252.1113	Positive
Methyl alpha-aspartylphenylalaninate	C14 H18 N2 O5	294.12214	Positive
trifluorbutane	C4 H7 F3	112.05051	Positive
5-Methoxy-1-(1-methyl-4-piperidinyl)-4-oxo-1,4-dihydro-2-pyridinecarboxylic acid	C13 H18 N2 O4	266.12706	Positive
Leu-arg	C12 H25 N5 O3	287.19616	Positive
beta-Ala-Phe	C12 H16 N2 O3	236.11649	Positive
Cadralazine	C12 H21 N5 O3	283.165	Positive
2-(4-Morpholinylmethyl)-3-(2-oxopropyl)-4(3H)-quinazolinone	C16 H19 N3 O3	301.14321	Positive
Ala-Tyr	C12 H16 N2 O4	252.11129	Positive
Lys-phe	C15 H23 N3 O3	293.17443	Positive
5-Methoxy-1-(1-methyl-4-piperidinyl)-4-oxo-1,4-dihydro-2-pyridinecarboxylic acid	C13 H18 N2 O4	266.12723	Positive
Val-Met	C10 H20 N2 O3 S	248.11984	Positive
N,N'-Di-Boc-guanidine	C11 H21 N3 O4	259.15371	Positive
Benzyl 4-(2-oxo-1-imidazolidinyl)-1-piperidinecarboxylate	C16 H21 N3 O3	303.15876	Positive
1,1',1''-Phosphinetriyltris(1H-imidazole)	C9 H9 N6 P	232.06285	Positive
Leu-arg	C12 H25 N5 O3	287.19623	Positive
Methyl 3-acetamido-6-[(3,6-diacetamidohexanoyl)amino]hexanoate	C19 H34 N4 O6	414.24828	Positive
Ethyl 1-(4-amino-6-oxo-1,6-dihydro-2-pyrimidinyl)-4-piperidinecarboxylate	C12 H18 N4 O3	266.13848	Positive
ala-met	C8 H16 N2 O3 S	220.08859	Positive
2-Methyl-2-propanyl 2-(4-methoxyphenyl)-4-(4-morpholinyl)-1H-imidazole-5-carboxylate	C19 H25 N3 O4	359.18568	Positive
O-Ethyl S-methyl methylphosphonothioate	C4 H11 O2 P S	154.02119	Positive
(2S)-3-Methyl-2-{[(2S,3R,4R,5R)-2,3,4,5,6-pentahydroxyhexyl]amino}butanoic acid	C11 H23 N O7	281.1462	Positive

Lys-Lys	C12 H26 N4 O3	274.20097	Positive
tert-butyl 3-[(N'-hydroxycarbamimidoyl)methyl]piperidine-1-carboxylate	C12 H23 N3 O3	257.1746	Positive
ile-pro-lys	C17 H32 N4 O4	356.24305	Positive
His-Leu	C12 H20 N4 O3	268.15398	Positive
tert-butyl 4-{[1-(hydroxyimino)-2-methylpropyl]carbamoyl}piperidine-1-carboxylate	C15 H27 N3 O4	313.20075	Positive
Di-Tert-Butyl 2-Oxo-1,8,11-Triazaspiro[5.6]Dodecane-8,11-Dicarboxylate	C19 H33 N3 O5	383.24277	Positive
asp-arg	C10 H19 N5 O5	289.13835	Positive
Leu-pro	C11 H20 N2 O3	228.14744	Positive
gln-leu-leu-pro	C22 H39 N5 O6	469.29089	Positive
7-(4-Methyl-1-piperazinyl)[1,3]thiazolo[4,5-d]pyrimidin-5-amine	C10 H14 N6 S	250.09901	Positive
Thr-val-leu	C15 H29 N3 O5	331.21135	Positive
(4-Cyclohexyl-1-piperazinyl)(4-nitrophenyl)methanone	C17 H23 N3 O3	317.17459	Positive
LYSINOALANINE, (S,R)-	C9 H19 N3 O4	233.13792	Positive
Methyl N-decanoylglycylprolyltryptophylleucinate	C35 H53 N5 O6	639.39663	Positive
Ethyl 4-[4-({2-[(3-ethoxy-3-oxo-1-phenylpropyl)amino]-2-oxoethyl}amino)-4-oxobutanoyl]-1-piperazinecarboxylate	C24 H34 N4 O7	490.24433	Positive
2-Amino-4-(methylsulfonyl)-N-(tetrahydro-2H-pyran-4-yl)butanamide	C10 H20 N2 O4 S	264.11467	Positive
Leu-Tyr	C15 H22 N2 O4	294.15852	Positive
Tris(trimethylsilyl)amine	C9 H27 N Si3	233.14598	Positive
4-[2-(4-Morpholinylmethyl)-2-nitrobutyl]morpholine	C13 H25 N3 O4	287.18504	Positive
Ethyl {[4-(dimethylamino)-6-(methoxyamino)-1,3,5-triazin-2-yl]oxy}acetate	C10 H17 N5 O4	271.12836	Positive
Lys-Pro	C11 H21 N3 O3	243.15904	Positive
1-BOC-ISONIPECOTIC ACID HYDRAZIDE	C11 H21 N3 O3	243.15874	Positive
Imazapyr	C13 H15 N3 O3	261.11181	Positive

1H-Imidazole-4,5-dicarbohydrazide	C5 H8 N6 O2	184.07155	Positive
Bis(2-methyl-2-propanyl) 2-carbamoyl-1,4-piperazinedicarboxylate	C15 H27 N3 O5	329.19559	Positive
(2R)-2-[(Ammonioacetyl)amino]-3-(4-hydroxyphenyl)propanoate	C11 H14 N2 O4	238.09568	Positive
FA-Gly-Nva-NH2	C14 H19 N3 O4	293.13817	Positive
Dibutyl(phenyl)phosphine sulfide	C14 H23 P S	254.12468	Positive
lys-thr-lys	C16 H33 N5 O5	375.24884	Positive
thr-arg	C10 H21 N5 O4	275.15974	Positive
MFCD11656061	C16 H29 N3 O6	359.2063	Positive
5-Oxoprolyltyrosinamide	C14 H17 N3 O4	291.12259	Positive
4-Methoxy-N-{[3-(2-pyrazinyl)-1H-1,2,4-triazol-5-yl]methyl}aniline	C14 H14 N6 O	282.12207	Positive
N-Hydroxy-2-[4-(N-hydroxycarbamimidoyl)phenyl]-1H-benzimidazole-5-carboximidamide	C15 H14 N6 O2	310.11687	Positive
ile-val-lys	C17 H34 N4 O4	358.25864	Positive
North-Methanocarbathymidine (**N**-**MCT**)	C12 H16 N2 O4	252.11131	Positive
3-Cyanopropyltriethoxysilane	C10 H21 N O3 Si	231.12988	Positive
2-Methyl-2-propanyl N-[(4-{[5-({[(2-methyl-2-propanyl)oxy]carbonyl}amino)pentyl]carbamoyl}-1H-imidazol-5-yl)carbonyl]glycinate	C21 H35 N5 O6	453.25983	Positive
Methyl [3-(3,4-diethoxyphenyl)-1H-1,2,4-triazol-1-yl]acetate	C15 H19 N3 O4	305.13811	Positive
val-ile-arg	C17 H34 N6 O4	386.26518	Positive
tert-Butyl (3Z)-3-amino-3-(hydroxyimino)propylcarbamate	C8 H17 N3 O3	203.12735	Positive
Methyl (2S,2aS,3a'R,4a'R,5aR,12bR,12b'R,12c'R,15aR,15a'S,17a'S)-12c'-hydroxy-10'-methoxy-2',3',4,4a',5,6,8,12c',13,13',14,14',17',17a'-tetradecahydro-4'H,5'H,16'H-spiro[furo[2',3':7,8]indolizino[8,1-cd]carbazole-2,6'-furo[2',3':7,8]indolizino[8,1-cd]pyrido[1,2,3-lm]carbazole]-7-carboxylate	C43 H50 N4 O6	718.37003	Positive
2-(4,5-Dihydro-4-methyl-4-(1-methylethyl)-5-oxo-1H-imidazol-2-yl)-5-(methoxymethyl)-3-pyridinecarboxylic acid	C15 H19 N3 O4	305.13811	Positive

1-Boc-3-(Amino)azetidine	C8 H16 N2 O2	172.12158	Positive
Biotinamidocaproyl hydrazide	C16 H29 N5 O3 S	371.19945	Positive
9-mercaptodethiobiotin	C10 H18 N2 O3 S	246.10429	Positive
Trialanine	C9 H17 N3 O4	231.12213	Positive
Yuanhuadin	C32 H42 O10	586.27604	Positive
2-(benzoylamino)-3-phenylpropanoic acid	C16 H15 N O3	269.1039	Positive
asn-trp-arg	C21 H30 N8 O5	474.23395	Positive
Leu-trp	C17 H23 N3 O3	317.17443	Positive
N,N'-Di-Boc-guanidine	C11 H21 N3 O4	259.15383	Positive
tert-Butyl 3-(((2-hydroxyethyl)thio)methyl)pyrrolidine-1-carboxylate	C12 H23 N O3 S	261.14101	Positive
Roblitinib	C25 H30 N8 O4	506.23833	Positive
(S)-4-(4-(5-(Aminomethyl)-2-oxooxazolidin-3-yl)phenyl)morpholin-3-one	C14 H17 N3 O4	291.12258	Positive
L-Prolyl-L-leucine	C20 H36 N6 O8	488.26001	Positive
(2S)-4-{[(Allyloxy)carbonyl]amino}-2-({[(2-methyl-2-propanyl)oxy]carbonyl}amino)butanoic acid	C13 H22 N2 O6	302.14675	Positive
6-{3-[(progesterone-4-yl)thiopropionyl]amino}hexanoic acid	C30 H45 N O5 S	531.30265	Positive
1,3,3-Trimethyl-6'-morpholinospiro[indoline-2,3'-naphtho[2,1-b][1,4]oxazine]	C26 H27 N3 O2	413.20922	Positive
tris(trimethylsilyl)phosphane	C9 H27 P Si3	250.11603	Positive
Methyl N-{[(2-methyl-2-propanyl)oxy]carbonyl}glycylprolylalanylthreoninate	C20 H34 N4 O8	458.23912	Positive
Dimepiperate	C7 H17 N7 O4	263.13466	Negative
febrifugin	C16 H19 N3 O3	301.14236	Negative
4-Nitrophenyl phenyl sulfide	C12 H9 N O2 S	231.0348	Negative
Lys-Asp-Trp	C21 H29 N5 O6	447.21122	Negative
6-[2-(Acetyloxymethyl)-4,5,6-trihydroxyoxan-3-yl]oxy-3,4,5-trihydroxyoxane-2-carboxylic acid	C14 H22 O13	398.10615	Negative

Table S8 displays the statistical significance of upregulated identified LC-MS metabolite levels determined by a volcano plot in Camp_48 vs. Cons1 in positive and negative mode. The names of the identified compounds, their molecular formula and molecular mass as well as the ionization mode in which the substance is detected are presented.

Table S8. LC/MS upregulated features that differed significantly between Camp_48 and Cons1 in ESI+ and ESI- mode. Data represents directed effected size for each bacteria obtained from volcano plot and significant at P-value ≤ 0.001.

Name	Formula	Molecular Weight	Ionization Mode
2,2-Dimethyl-4-oxo-3,6,9,12-tetraoxa-5-azatetradecan-14-oic acid	C11 H21 N O7	279.13218	Positive
3-(3,4-Dimethoxyphenyl)-1-ethyl-6-methylpyrimido[5,4-e][1,2,4]triazine-5,7(1H,6H)-dione	C16 H17 N5 O4	343.12712	Positive
Methyl 4,6-dideoxy-4-[(2,4-dihydroxybutanoyl)amino]hexopyranoside	C11 H21 N O7	279.13145	Negative

Table S9 displays the statistical significance of downregulated identified LC-MS metabolite levels determined by a volcano plot in Camp_48 vs. Cons1 in positive and negative mode. The names of the identified compounds, their molecular formula and molecular mass as well as the ionization mode in which the substance is detected are presented.

Table S9. LC/MS downregulated features that differed significantly between Camp_48 and Cons1 in ESI+ and ESI- mode. Data represents directed effected size for each bacteria obtained from volcano plot and significant at P-value ≤ 0.001.

Name	Formula	Molecular Weight	Ionization Mode
Prolylleucine (Pro-Leu)	C11 H20 N2 O3	228.14763	Positive
REPOSAL	C14 H18 N2 O3	262.132	Positive
Leu-Pro	C11 H20 N2 O3	228.14774	Positive
2-[(6,6-Dimethyl-2-morpholinyl)methoxy]-N,N-dimethylacetamide	C11 H22 N2 O3	230.16337	Positive
Leu-Leu	C12 H24 N2 O3	244.17901	Positive
Val-pro	C10 H18 N2 O3	214.13205	Positive
Val-Leu	C11 H22 N2 O3	230.16335	Positive
Leu-Ile (leucine - isoleucine)	C12 H24 N2 O3	244.17869	Positive
N2-Acetyl-L-lysine	C8 H16 N2 O3	188.11638	Positive

2-Nitroso-6,7-dimethoxytetrahydroisoquinoline	C11 H14 N2 O3	222.10073	Positive
met-val	C10 H20 N2 O3 S	248.11979	Positive
[4-(Aminomethyl)hexahydro-1H-[1,4]oxazino[3,4-c][1,4]oxazin-4-yl]methanol	C9 H18 N2 O3	202.13218	Positive
2-(Trifluoromethyl)-5-pyrimidinylboronic acid	C5 H4 B F3 N2 O2	192.03149	Positive
2,2'-[(6-Aminohexyl)imino]diacetic acid	C10 H20 N2 O4	232.14266	Positive
3,5-Dimethoxy-N-[2-(4-morpholinyl)ethyl]benzamide	C15 H22 N2 O4	294.15855	Positive
9-mercaptodethiobiotin	C10 H18 N2 O3 S	246.10421	Positive
trifluorbutane	C4 H7 F3	112.05051	Positive
Methyl 3-amino-3-(3-pyridinyl)propanoate	C9 H12 N2 O2	180.09023	Positive
5-Methoxy-1-(1-methyl-4-piperidinyl)-4-oxo-1,4-dihydro-2-pyridinecarboxylic acid	C13 H18 N2 O4	266.12706	Positive
Leu-arg	C12 H25 N5 O3	287.19616	Positive
beta-Ala-Phe	C12 H16 N2 O3	236.11649	Positive
1-Deoxynojirimycin	C6 H13 N O4	163.08472	Positive
2-(4-Morpholinylmethyl)-3-(2-oxopropyl)-4(3H)-quinazolinone	C16 H19 N3 O3	301.14321	Positive
3-(3-Ethoxy-4-hydroxy-1-pyrrolidinyl)propanoic acid	C9 H17 N O4	203.11616	Positive
Ala-Tyr	C12 H16 N2 O4	252.11129	Positive
Lys-phe	C15 H23 N3 O3	293.17443	Positive
N-{[(2-Methyl-2-propanyl)oxy]carbonyl}-L-isoleucyl-L-glutamine	C16 H29 N3 O6	359.20631	Positive
4,4'-Dicyanobenzhydrol	C15 H10 N2 O	234.07838	Positive
Benzyl 4-(2-oxo-1-imidazolidinyl)-1-piperidinecarboxylate	C16 H21 N3 O3	303.15876	Positive
1,1',1''-Phosphinetriyltris(1H-imidazole)	C9 H9 N6 P	232.06285	Positive
4-Methyl-1H-[1,2,3]triazolo[4,5-d]pyridazin-7-ol	C5 H5 N5 O	151.04967	Positive
Ileu-arg (isoleucine - arginine)	C12 H25 N5 O3	287.19623	Positive
Methyl 3-acetamido-6-[(3,6-diacetamidohexanoyl)amino]hexanoate	C19 H34 N4 O6	414.24828	Positive

O-Ethyl S-methyl methylphosphonothioate	C4 H11 O2 P S	154.02119	Positive
5,6-Diamino-2-thiouracil	C4 H6 N4 O S	158.02586	Positive
(3-Amino-1-azetidinyl)(4-methyl-1,2,3-thiadiazol-5-yl)methanone	C7 H10 N4 O S	198.05725	Positive
tert-butyl 4-{[1-(hydroxyimino)-2-methylpropyl]carbamoyl}piperidine-1-carboxylate	C15 H27 N3 O4	313.20075	Positive
(4-Cyclohexyl-1-piperazinyl)(4-nitrophenyl)methanone	C17 H23 N3 O3	317.17459	Positive
Methyl N-decanoylglycylprolyltryptophylleucinate	C35 H53 N5 O6	639.39663	Positive
[(4-Amino-5-propyl-4H-1,2,4-triazol-3-yl)sulfanyl]acetic acid	C7 H12 N4 O2 S	216.06781	Positive
N-(3-Methyl-2-buten-1-yl)-2-(methylsulfanyl)-7H-purin-6-amine	C11 H15 N5 S	249.10516	Positive
N-{(2R)-2-[2-(Hydroxyamino)-2-oxoethyl]-4-methylpentanoyl}-3-methyl-L-valyl-N-(2-aminoethyl)-L-alaninamide	C19 H37 N5 O5	415.28018	Positive
Tris(trimethylsilyl)amine	C9 H27 N Si3	233.14598	Positive
4-[2-(4-Morpholinylmethyl)-2-nitrobutyl]morpholine	C13 H25 N3 O4	287.18504	Positive
2-(3,5-Dioxomorpholino)acetic acid	C6 H7 N O5	173.03186	Positive
2-(4-Isopropyl-4-methyl-5-oxo-2-imidazolin-2-yl)nicotinic acid	C13 H15 N3 O3	261.11181	Positive
1-[1-(Methylsulfonyl)-3-pyrrolidinyl]-1H-1,2,3-triazole-4-carbaldehyde	C8 H12 N4 O3 S	244.06276	Positive
2-[(1,2-Dimethyl-4-nitro-1H-imidazol-5-yl)amino]ethanol	C7 H12 N4 O3	200.0903	Positive
Bis(2-methyl-2-propanyl) 2-carbamoyl-1,4-piperazinedicarboxylate	C15 H27 N3 O5	329.19559	Positive
FA-Gly-Nva-NH2	C14 H19 N3 O4	293.13817	Positive
N-Ethyl-N'-isopropyl-6-(4-morpholinyl)-1,3,5-triazine-2,4-diamine	C12 H22 N6 O	266.1862	Positive
6-(4-(Methylsulfonyl)piperazin-1-yl)pyridin-3-amine	C10 H16 N4 O2 S	256.09917	Positive
N-Hydroxy-2-[4-(N-hydroxycarbamimidoyl)phenyl]-1H-benzimidazole-5-carboximidamide	C15 H14 N6 O2	310.11687	Positive
2-(2-AMINOETHYLAMINO)-5-NITROPYRIDINE	C7 H10 N4 O2	182.07987	Positive
NP-001346	C11 H15 N5 O3 S	297.09016	Positive
3-(3-Ethoxy-4-nitro-1H-pyrazol-1-yl)-1-propanamine	C8 H14 N4 O3	214.10598	Positive

L-alpha-Aspartyl-L-phenylalanine	C13 H16 N2 O5	280.10647	Positive
5-Amino-3-(methylsulfanyl)-1H-pyrazole-4-carboxamide	C5 H8 N4 O S	172.04159	Positive
3-Cyanopropyltriethoxysilane	C10 H21 N O3 Si	231.12988	Positive
2-Nitrophenyl 5-{1-[4-(2-methyl-2-propanyl)benzoyl]-2-oxohexahydro-1H-thieno[3,4-d]imidazol-4-yl}pentanoate	C27 H31 N3 O6 S	525.19297	Positive
2-Methyl-2-propanyl N-[(4-{[5-({[(2-methyl-2-propanyl)oxy]carbonyl}amino)pentyl]carbamoyl}-1H-imidazol-5-yl)carbonyl]glycinate	C21 H35 N5 O6	453.25983	Positive
LY2090314	C28 H25 F N6 O3	512.19846	Positive
Methyl (2S,2aS,3a'R,4a'R,5aR,12bR,12b'R,12c'R,15aR,15a'S,17a'S)-12c'-hydroxy-10'-methoxy-2',3',4,4a',5,6,8,12c',13,13',14,14',17',17a'-tetradecahydro-4'H,5'H,16'H-spiro[furo[2',3':7,8]indolizino[8,1-c d]carbazole-2,6'-furo[2',3':7,8]indolizino[8,1-cd]pyrido[1,2,3-lm]carbazole]-7-carboxylate	C43 H50 N4 O6	718.37003	Positive
2-(4,5-Dihydro-4-methyl-4-(1-methylethyl)-5-oxo-1H-imidazol-2-yl)-5-(methoxymethyl)-3-pyridinecarboxylic acid	C15 H19 N3 O4	305.13811	Positive
N-(2-Azidoethyl)-N-{2-[{2-[methyl(propionyl)amino]ethyl}(propionyl)amino]ethyl}propanamide	C16 H30 N6 O3	354.23857	Positive
4-Methyl-1H-[1,2,3]triazolo[4,5-d]pyridazin-7-ol	C5 H5 N5 O	151.04971	Positive
1-Azido-2-{2-[2-(2-isothiocyanatoethoxy)ethoxy]ethoxy}ethane	C9 H16 N4 O3 S	260.09403	Positive
2-(Hydroxymethyl)-2-(1H-tetrazol-1-yl)-1,3-propanediol	C5 H10 N4 O3	174.0746	Positive
Iridodial glucoside tetraacetate	C24 H34 O11	498.21039	Positive
4-[4-(3-Methoxypropyl)-5-methyl-4H-1,2,4-triazol-3-yl]-1-methyl-1H-pyrazol-5-amine	C11 H18 N6 O	250.15479	Positive
ile-ile-lys	C18 H36 N4 O4	372.27443	Positive
N,N'-Di-Boc-guanidine	C11 H21 N3 O4	259.15383	Positive
Thr-val-leu	C15 H29 N3 O5	331.21119	Positive
L-Leucyl-L-alanyl-L-proline	C14 H25 N3 O4	299.18503	Positive
1-[4-(1H-Purin-6-yl)-2-morpholinyl]methanamine	C10 H14 N6 O	234.12351	Positive
ala-leu-ala-pro	C17 H30 N4 O5	370.22215	Positive

Iridodial glucoside tetraacetate	C24 H34 O11	498.20809	Positive
(3beta,16alpha,22alpha)-16,28-Dihydroxy-22-{[(2Z)-2-methyl-2-butenoyl]oxy}olean-12-en-3-yl beta-D-galactopyranosyl-(1->2)-[beta-D-xylopyranosyl-(1->2)-alpha-L-arabinopyranosyl-(1->3)]-beta-D-glucopyra nosiduronic acid	C57 H90 O24	1158.58213	Positive
3-(4-Pyridinyl)-5H-indeno[1,2-c]pyridazin-5-one	C16 H9 N3 O	259.0739	Positive
Dimepiperate	C7 H17 N7 O4	263.13466	Negative
3-hydroxydodecanoylcarnitine	C19 H37 N O5	359.26677	Negative
His-Leu	C12 H20 N4 O3	268.15309	Negative
N-(PHENYLMETHYLIDENE)BENZENESULFONAMIDE	C13 H11 N O2 S	245.05056	Negative
Glaurin	C16 H32 O4	288.22986	Negative
Myriocin	C21 H39 N O6	401.27722	Negative
4-Nitrophenyl phenyl sulfide	C12 H9 N O2 S	231.0348	Negative
Ethyl 2-(benzoylamino)-7-oxo-4,5,6,7-tetrahydro-1-benzothiophene-3-carboxylate	C18 H17 N O4 S	343.08754	Negative
N,N′-Di-Boc-S-methylisothiourea	C12 H22 N2 O4 S	290.13007	Negative
val-leu-gly-lys	C19 H37 N5 O5	415.27912	Negative
5-Oxidanidyl-5-oxidanylidenenorvalylarginine	C11 H20 N5 O5	302.14751	Negative
4-Methyl-2-{(E)-[4-(methylsulfanyl)benzylidene]amino}phenol	C15 H15 N O S	257.08702	Negative
6-[2-(Acetyloxymethyl)-4,5,6-trihydroxyoxan-3-yl]oxy-3,4,5-trihydroxyoxane-2-carboxylic acid	C14 H22 O13	398.10615	Negative
9-Oxo-9,10-dihydro-2-acridinesulfonic acid	C13 H9 N O4 S	275.02508	Negative
Dimethyl(2-methyl-2-propanyl)silyl N-[dimethyl(2-methyl-2-propanyl)silyl]isoleucinate	C18 H41 N O2 Si2	359.2667	Negative

Table S10 displays the statistical significance of upregulated identified LC-MS metabolite levels determined by a volcano plot in Camp_83 vs. Cons1 in positive and negative mode. The names of the identified compounds, their molecular formula and molecular mass as well as the ionization mode in which the substance is detected are presented.

Table S10. LC/MS upregulated features that differed significantly between Camp_83 and Cons1 in ESI+ and ESI- mode. Data represents directed effected size for each bacteria obtained from volcano plot and significant at P-value ≤ 0.001.

Name	Formula	Molecular Weight	Ionization Mode
REPOSAL	C14 H18 N2 O3	262.132	Positive
Prolylleucine (Pro-Leu)	C11 H20 N2 O3	228.14774	Positive
tert-Butyl N-[1-(aminocarbonyl)-3-methylbutyl]carbamate	C11 H22 N2 O3	230.16344	Positive
2-(2-amino-3-methylbutanamido)-3-phenylpropanoic acid	C14 H20 N2 O3	264.14782	Positive
N2-Acetyl-L-lysine	C8 H16 N2 O3	188.11638	Positive
Leu-Tyr	C15 H22 N2 O4	294.15839	Positive
3-{[(4-Methoxy-1-piperidinyl)carbonyl]amino}benzoic acid	C14 H18 N2 O4	278.12713	Positive
N-(3-Nitrophenyl)octanamide	C14 H20 N2 O3	264.14786	Positive
tert-Butyl hydrazodiformate	C10 H20 N2 O4	232.14262	Positive
N-Boc-serinol	C8 H17 N O4	191.11599	Positive
val-arg	C11 H23 N5 O3	273.18047	Positive
Val-Gln	C10 H19 N3 O4	245.13791	Positive
[4-(Aminomethyl)hexahydro-1H-[1,4]oxazino[3,4-c][1,4]oxazin-4-yl]methanol	C9 H18 N2 O3	202.13215	Positive
Cadralazine	C12 H21 N5 O3	283.165	Positive
Lys-phe	C15 H23 N3 O3	293.17443	Positive
Val-Met	C10 H20 N2 O3 S	248.11984	Positive
N,N'-Di-Boc-guanidine	C11 H21 N3 O4	259.15371	Positive
Ethyl 1-(4-amino-6-oxo-1,6-dihydro-2-pyrimidinyl)-4-piperidinecarboxylate	C12 H18 N4 O3	266.13848	Positive
ala-met	C8 H16 N2 O3 S	220.08859	Positive
Lys-Lys	C12 H26 N4 O3	274.20097	Positive

tert-butyl 3-[(N'-hydroxycarbamimidoyl)methyl]piperidine-1-carboxylate	C12 H23 N3 O3	257.1746	Positive
His-Leu	C12 H20 N4 O3	268.15398	Positive
asp-arg	C10 H19 N5 O5	289.13835	Positive
Leu-pro	C11 H20 N2 O3	228.14744	Positive
7-(4-Methyl-1-piperazinyl)[1,3]thiazolo[4,5-d]pyrimidin-5-amine	C10 H14 N6 S	250.09901	Positive
Ethyl 4-[4-({2-[(3-ethoxy-3-oxo-1-phenylpropyl)amino]-2-oxoethyl}amino)-4-oxobutanoyl]-1-piperazinecarboxylate	C24 H34 N4 O7	490.24433	Positive
Leu-Tyr	C15 H22 N2 O4	294.15852	Positive
Tris(trimethylsilyl)amine	C9 H27 N Si3	233.14598	Positive
Lys-Pro	C11 H21 N3 O3	243.15904	Positive
1H-Imidazole-4,5-dicarbohydrazide	C5 H8 N6 O2	184.07155	Positive
Dibutyl(phenyl)phosphine sulfide	C14 H23 P S	254.12468	Positive
thr-arg	C10 H21 N5 O4	275.15974	Positive
4-Methoxy-N-{[3-(2-pyrazinyl)-1H-1,2,4-triazol-5-yl]methyl}aniline	C14 H14 N6 O	282.12207	Positive
North-Methanocarbathymidine (**N**-**MCT**)	C12 H16 N2 O4	252.11131	Positive
val-ile-arg	C17 H34 N6 O4	386.26518	Positive
asn-trp-arg	C21 H30 N8 O5	474.23395	Positive
N,N'-Di-Boc-guanidine	C11 H21 N3 O4	259.15383	Positive
Roblitinib	C25 H30 N8 O4	506.23833	Positive
(S)-4-(4-(5-(Aminomethyl)-2-oxooxazolidin-3-yl)phenyl)morpholin-3-one	C14 H17 N3 O4	291.12258	Positive
6-{3-[(progesterone-4-yl)thiopropionyl]amino}hexanoic acid	C30 H45 N O5 S	531.30265	Positive
1,3,3-Trimethyl-6'-morpholinospiro[indoline-2,3'-naphtho[2,1-b][1,4]oxazine]	C26 H27 N3 O2	413.20922	Positive

Table S11 displays the statistical significance of downregulated identified LC-MS metabolite levels determined by a volcano plot in Camp_83 vs. Cons1 in positive and negative mode. The names of the identified compounds, their molecular formula and molecular mass as well as the ionization mode in which the substance is detected are presented.

Table S11. LC/MS downregulated features that differed significantly between Camp_83 and Cons1 in ESI+ and ESI- mode. Data represents directed effected size for each bacteria obtained from volcano plot and significant at P-value ≤ 0.001.

Name	Formula	Molecular Weight	Ionization Mode
N-Acetyl-L-leucine	C8 H15 N O3	173.1056	Positive
1-Deoxynojirimycin	C6 H13 N O4	163.08472	Positive
4-Methyl-1H-[1,2,3]triazolo[4,5-d]pyridazin-7-ol	C5 H5 N5 O	151.04967	Positive
2,2'-(1,1-Cyclopropanediyl)bis(4,4,5,5-tetramethyl-1,3,2-dioxaborolane)	C15 H28 B2 O4	294.21767	Positive
N-(3-Methyl-2-buten-1-yl)-2-(methylsulfanyl)-7H-purin-6-amine	C11 H15 N5 S	249.10516	Positive
2-(3,5-Dioxomorpholino)acetic acid	C6 H7 N O5	173.03186	Positive
N-Ethyl-N'-isopropyl-6-(4-morpholinyl)-1,3,5-triazine-2,4-diamine	C12 H22 N6 O	266.1862	Positive
Palmitelaidic acid	C16 H30 O2	254.22493	Positive
N-(2-Azidoethyl)-N-{2-[{2-[methyl(propionyl)amino]ethyl}(propionyl)amino]ethyl}propanamide	C16 H30 N6 O3	354.23857	Positive
1-Azido-2-{2-[2-(2-isothiocyanatoethoxy)ethoxy]ethoxy}ethane	C9 H16 N4 O3 S	260.09403	Positive
2-(Hydroxymethyl)-2-(1H-tetrazol-1-yl)-1,3-propanediol	C5 H10 N4 O3	174.0746	Positive
Iridodial glucoside tetraacetate	C24 H34 O11	498.21039	Positive
N-(7-Amino-6-methyl-7H-[1,2,4]triazolo[4,3-b][1,2,4]triazol-3-yl)acetamide	C6 H9 N7 O	195.08761	Positive
L-Leucyl-L-alanyl-L-proline	C14 H25 N3 O4	299.18503	Positive
1-[4-(1H-Purin-6-yl)-2-morpholinyl]methanamine	C10 H14 N6 O	234.12351	Positive

ala-leu-ala-pro	C17 H30 N4 O5	370.22215	Positive
Iridodial glucoside tetraacetate	C24 H34 O11	498.20809	Positive
N~1~-[3-(Diethylamino)propyl]-4-(1-piperidinylsulfonyl)-1,2-benzenediamine	C18 H32 N4 O2 S	368.2245	Positive
3-hydroxydodecanoylcarnitine	C19 H37 N O5	359.26677	Negative
His-Leu	C12 H20 N4 O3	268.15309	Negative
N-(PHENYLMETHYLIDENE)BENZENESULFONAMIDE	C13 H11 N O2 S	245.05056	Negative
Glaurin	C16 H32 O4	288.22986	Negative
Myriocin	C21 H39 N O6	401.27722	Negative
Ethyl 2-(benzoylamino)-7-oxo-4,5,6,7-tetrahydro-1-benzothiophene-3-carboxylate	C18 H17 N O4 S	343.08754	Negative
val-leu-gly-lys	C19 H37 N5 O5	415.27912	Negative
5-Oxidanidyl-5-oxidanylidenenorvalylarginine	C11 H20 N5 O5	302.14751	Negative
Dimethyl(2-methyl-2-propanyl)silyl N-[dimethyl(2-methyl-2-propanyl)silyl]isoleucinate	C18 H41 N O2 Si2	359.2667	Negative
